# Hypoxia-Inducible Factors (HIFs) and Phosphorylation: Impact on Stability, Localization, and Transactivity

**DOI:** 10.3389/fcell.2016.00011

**Published:** 2016-02-23

**Authors:** Thomas Kietzmann, Daniela Mennerich, Elitsa Y. Dimova

**Affiliations:** Faculty of Biochemistry and Molecular Medicine, Biocenter Oulu, University of OuluFinland

**Keywords:** phosphorylation, HIF-1α, hypoxia, kinase, MAPK pathway, PI3K/PKB pathway

## Abstract

The hypoxia-inducible factor α-subunits (HIFα) are key transcription factors in the mammalian response to oxygen deficiency. The HIFα regulation in response to hypoxia occurs primarily on the level of protein stability due to posttranslational hydroxylation and proteasomal degradation. However, HIF α-subunits also respond to various growth factors, hormones, or cytokines under normoxia indicating involvement of different kinase pathways in their regulation. Because these proteins participate in angiogenesis, glycolysis, programmed cell death, cancer, and ischemia, HIFα regulating kinases are attractive therapeutic targets. Although numerous kinases were reported to regulate HIFα indirectly, direct phosphorylation of HIFα affects HIFα stability, nuclear localization, and transactivity. Herein, we review the role of phosphorylation-dependent HIFα regulation with emphasis on protein stability, subcellular localization, and transactivation.

## Introduction

An adaequate supply of oxygen is mandatory for aerobic life. To cope with an inadequate O_2_ supply, commonly termed hypoxia, mammals have developed response mechanisms which are crucial for their survival.

To achieve responsiveness toward hypoxia on the molecular level, cells integrate a complex biochemical system involving short-term reactions/modifications with no changes in gene expression and a long-term programme including changes in gene expression. Both processes can be interlinked; in particular, when the short-term response includes changes in the activity of enzymes which initiate a series of posttranslational signaling events that often regulate the activity of transcription factors and thus gene expression. On the level of gene expression the response to hypoxia is crucially dependent on the α-subunits of hypoxia-inducible transcription factors (HIFα) (Semenza, 2003; Kaelin, 2011; Masson and Ratcliffe, 2014).

As such, HIF α-subunit proteins contribute to proper embryonic development and to the pathology of many diseases associated with hypoxia like anemia, myocardial infarction, thrombosis, atherosclerosis, diabetes mellitus, or cancer (Semenza, 2003; Kaelin, 2011; Masson and Ratcliffe, 2014).

To achieve adaequate function, HIFα levels, subcellular distribution and activity need to be tightly regulated. Although regulation at the transcriptional and translational level was shown to play a role, posttranslational stabilization of HIFα proteins in response to hypoxia appears to be of major importance (Wenger, 2002; Gross et al., 2003; Gorlach, 2009; Kietzmann, 2009).

**Figure 1:**
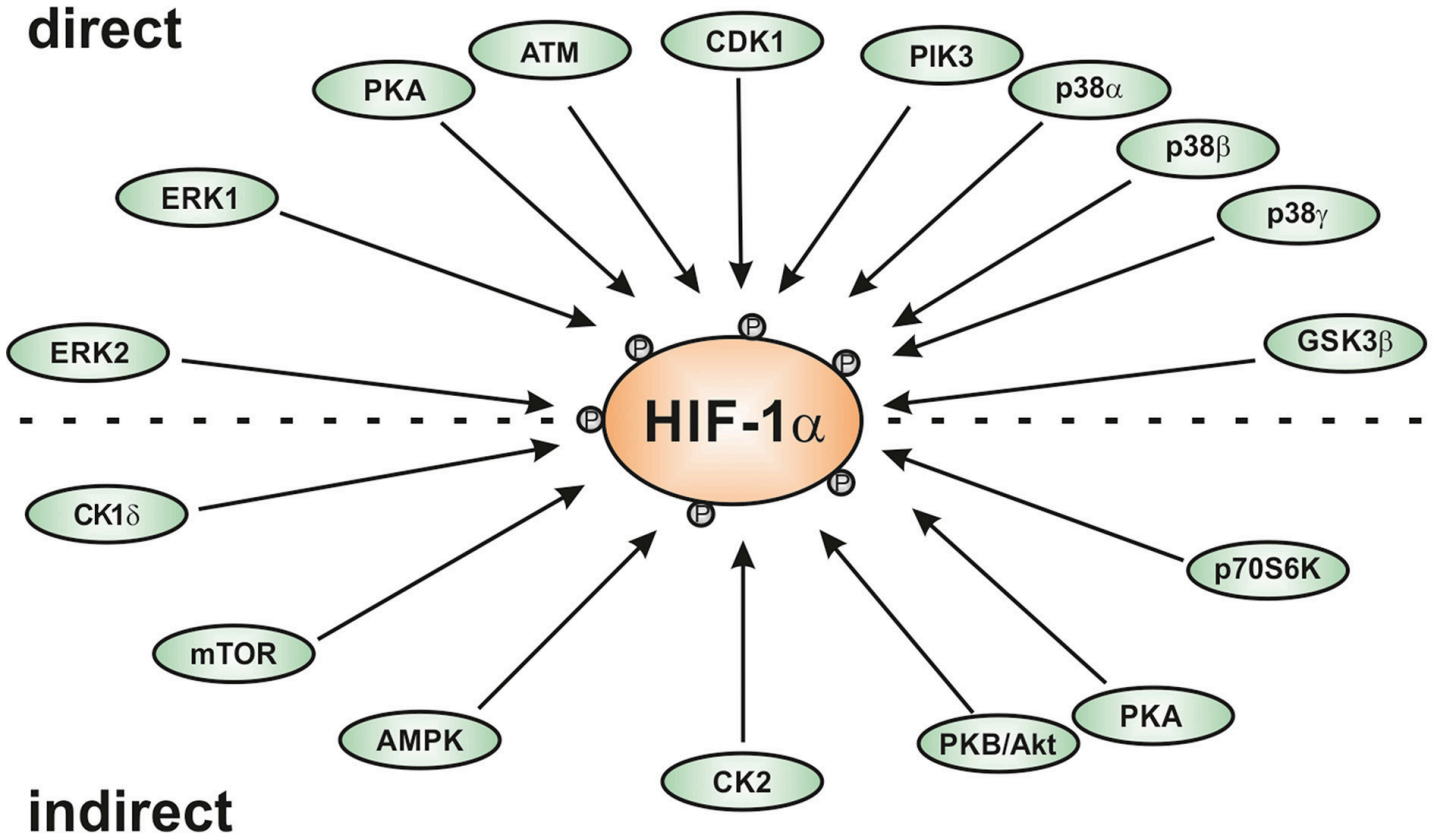
**Scheme of kinases involved in regulating HIF-1α either direcly or indirectly**. AMPK, AMP-activated kinase; ATM, ataxia and teleangiectasia mutated; CK1, casein kinase1; CDK1, cyclin-dependent kinase-1; ERK; extracellular regulated kinase; GSK3β, glycogen synthase kinase-3β; PKA, protein kinase A; PKB/Akt, protein kinase B or Akt kinase; p38, p38 mitogen activated protein kinase; Plk3, polo-like kinase-3.

Interestingly, the HIFα proteins are not only regulated by hypoxia, but also in response to various stresses, growth and coagulation factors, hormones, or cytokines under normoxic conditions (reviewed by Dimova et al., 2009). These “normoxic” HIFα stimuli often use different protein kinase regulated pathways for signal transduction indicating an important role of different kinases in HIFα regulation. Indeed, different kinases have been identified to regulate HIFα in a direct or indirect fashion (Figure 1). This review will primarily discuss the role of the kinases using HIFα proteins as a direct substrate and the impact of these modifications on HIFα stabilization, nuclear translocation, and transactivation.

## Hypoxia-inducible transcription factors: α- and β-subunits

Three O_2_-sensitive HIFα proteins (HIF-1α, HIF-2α -also known as EPAS (Tian et al., 1997), HLF (Ema et al., 1997), HRF (Flamme et al., 1997), or MOP2 (Hogenesch et al., 1998)—and HIF-3α) are known today. Together with HIF β-subunits, primarily represented by the stable nuclear and ubiquitously found ARNT (arylhydrocarbon receptor-nuclear translocator) protein, they form heterodimeric transcription factors binding to hypoxia response elements (HRE) with the core DNA sequence 5′-RCGTG-3′ (Wenger et al., 2005).

The best studied HIFα isoforms are HIF-1α and HIF-2α which share a number of structural and functional similarities but also show some differences with respect to cell type expression pattern, embryonic deletion phenotypes, target genes, and effects during carcinogenesis (Hu et al., 2003; Scortegagna et al., 2003; Sowter et al., 2003). Not much is known about HIF-3α from which several splice variants exist in humans (Pasanen et al., 2010) and where some variants as well as a mouse splice variant termed inhibitory PAS protein (IPAS) appear to act as negative regulators of the hypoxic response (Makino et al., 2007; Heikkila et al., 2011) while others appear to act as an oxygen-regulated transcription activator (for review see Duan, 2015).

**Figure 2:**
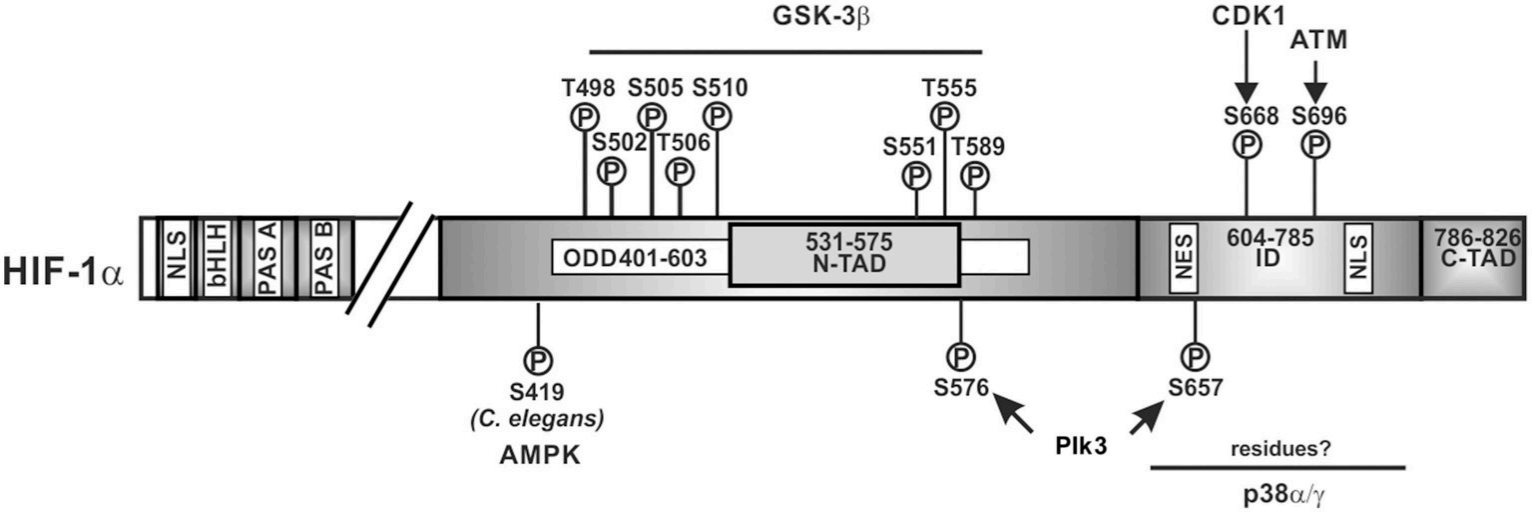
**Kinases and amino acid residues in HIF-1α involved in regulation of HIF-1α stability**. Scheme of HIF-1α and its domain organization with specific amino acid residues which can be phosphorylated; all have been shown to contribute to the regulation of HIF-1α protein stability. HIF-1α is phosphorylated on specific residues (T498, S502, S505, T506, and S510 or S551, T555, and S589) by GSK3β; Plk3 can phosphorylate S576 and S657; ATM can phosphorylate S696 and CDK1 can phosphorylate S668. ATM, ataxia and teleangiectasia mutated; CDK1, cyclin-dependent kinase-1; GSK3β, glycogen synthase kinase-3b; Plk3, polo-like kinase 3; bHLH, basic helix loop helix domain; NLS, nuclear localization sequence; PAS, Per-ARNT-Sim domain; N-TAD, N-terminal transactivation domain; ID, inhibitory domain; C-TAD, C-terminal transactivation domain; numbers indicate the amino acid residue range of the respective domain.

Like the ARNT proteins, the HIF α-proteins belong to the basic helix-loop-helix (bHLH) PAS (Per-ARNT-Sim) protein family (Wang et al., 1995a); HIF-1α and HIF-2α show the highest degree of sequence identity in the bHLH (~85%), PAS-A (~68%), and PAS-B (~73%) domains. Both contain also two conserved nuclear localization sequences (NLS) responsible for translocation to the nucleus; they are localized in the N-terminus (aa 17–33 in HIF-1α; aa 1–50 in HIF-2α) and in the C-terminus (aa 718–721 in HIF-1α; aa 689–870 in HIF-2α) (Kallio et al., 1998). Except for the full length HIF-3α which does not contain a C-terminal transactivation domain but a unique LZIP (leucine zipper) C-terminal domain (Hara et al., 2001; Kietzmann et al., 2001), HIF α-subunits contain also a N-terminal transactivation domain (N-TAD) and a C-terminal transactivation domain (C-TAD). An oxygen-dependent degradation domain (ODDD, aa 401–603 in HIF-1α; aa 517–682 in HIF-2α) is overlapping the N-TAD and is important for the oxygen-dependent regulation of all vertebrate HIFα proteins (Huang et al., 1998; Duan, 2015). The residues between the N-TAD and C-TAD constitute an inhibitory domain (ID) (Jiang et al., 1997) (Figure 2).

## Hypoxia-inducible regulation of α-subunits

The levels of the HIF α-subunits increase exponentially with declining O_2_ concentration as a result of reduced hydroxylation, ubiquitylation and proteasomal degradation (Semenza, 2003; Kaelin, 2011; Masson and Ratcliffe, 2014). To date, four HIF specific prolyl 4-hydroxylase domain containing enzymes (PHDs) have been identified from which PHD2 appears to be of major importance for HIFα degradation (Berra et al., 2003). All HIF hydroxylases belong to a family of dioxygenases which depend on the presence of O_2_ for their action. Thus, in the presence of O_2_, i.e., normoxia, PHDs are able to hydroxylate crucial proline residues in the HIFα ODDDs (P402/P564 in HIF-1α; P405/P531 in HIF-2α; P492 in HIF-3α). This event recruits the von Hippel-Lindau tumor suppressor protein (pVHL) which together with Elongin C, Elongin B, RBX1, Cullin 2, and an E2 ubiquitin-conjugating enzyme forms an ubiquitin E3 ligase complex. As a consequence, HIFα proteins become ubiquitylated and degraded by the proteosome (Semenza, 2003; Kaelin, 2011; Masson and Ratcliffe, 2014).

Another hydroxylase called factor-inhibiting HIF (FIH-1) hydroxylates an asparagine in the C-TADs of HIF-1α and HIF-2α (N803 in HIF-1α and N847 in HIF-2α) with the result that the interaction of the HIFα proteins with the co-activators CBP/p300 is inhibited (Mahon et al., 2001; Hewitson et al., 2002; Lando et al., 2002). Thus, a limited O_2_ supply decreases the activities of HIF hydroxylases and allows HIFα stabilization, followed by nuclear translocation, dimerization, and transactivation (for review see Kaelin, 2005).

## Regulation of HIF α-subunits by phosphorylation

Phosphorylation is a crucial posttranslational modification which regulates the activity and stability of various proteins including transcription factors. However, the extent to which transcription factors including HIFα proteins are phosphorylated may vary according to the signal, cell-type, or tissue. Thus, it is plausible that a modulation of HIFα action due to phosphorylation may be a cell type specific event which could be explained by different layers of regulations where kinases are affected depending on the cellular context.

The first evidence indicating that phosphorylation plays a role in HIFα regulation came from electrophoretic mobility shift assay experiments where addition of calf intestinal alkaline phosphatase to hypoxic nuclear extracts led to a loss of HIF-1 DNA-binding activity (Wang et al., 1995b). In the meantime a panel of protein kinases was reported to affect HIFα regulation, mainly HIF-1α, indirectly or directly (for review see Dimova et al., 2009). Thereby it appeared that direct phosphorylation of HIFα has an immediate impact on HIFα stability, nuclear localization, transactivity, and protein-protein interactions.

### Phosphorylation of HIFα proteins: role for subunit stabilization

A number of findings indicated that the PI3K/PKB(Akt) pathway can induce HIFα, transcription, stabilization (Mazure et al., 1997; Zhong et al., 2000; Zundel et al., 2000; Hirota and Semenza, 2001), translation (Koritzinsky et al., 2006), and coactivator recruitment (Kallio et al., 1998). So far, no evidence has been presented showing that HIFα proteins are directly phosphorylated by PKB(Akt); rather its action is indirect involving other PKB/Akt targets. Although a number of PKB/Akt targets are known, so far only the human homolog of mouse double minute-2 (HDM2) (Bardos et al., 2004; Skinner et al., 2004), mammalian target of rapamycin (mTOR) (Treins et al., 2002), and glycogen synthase kinase-3 (GSK3) (Flügel et al., 2007, 2012) were shown to affect HIF-1α levels with most evidence indicating that only GSK3 acts directly on HIF-1α.

Although the name GSK3 implies that this is a specific kinase acting only on glycogen synthase, it is rather pleiotropic with a number of substrates through which GSK3 may affect various signaling pathways often associated with hypoxia like developmental processes, stem cell renewal, cell proliferation, and apoptosis (reviewed in Cohen and Frame, 2001; Grimes and Jope, 2001; Force and Woodgett, 2009).

Mammals possess two GSK3 isoforms, GSK3α (51 kDa) and GSK3β (47 kDa) which are structurally similar, but not entirely functionally overlapping (reviewed in Force and Woodgett, 2009). This became evident from the different phenotypes of GSK3 knockout mice. GSK3β^−∕−^ mice are embryonically lethal and die around day 16 because of hepatic apoptosis and a cardiac pattern defect (Hoeflich et al., 2000; Kerkela et al., 2008). By contrast, GSK3α^−∕−^ mice are viable, and fertile (MacAulay et al., 2007). Interestingly, it exists also a minor spliced GSK3β variant called GSK3β2 that contains a 13-amino acid residue insert within the kinase domain. This isoform was shown to be neuron-specific and has reduced kinase activity toward the microtubule-associated protein, tau, compared to GSK3β (Mukai et al., 2002; Saeki et al., 2011).

GSK3 is a target of the PKB/Akt pathway and it is unusual that its protein kinase activity tends to be high in resting cells. Furthermore, its inhibition is mediated by various stimuli, such as growth factors, cytokines, and hormones. PKB/Akt can phosphorylate both GSK3 isoforms (S21 of GSK3α and S9 of GSK3β), leading to an inhibition of GSK3 activity (Cross et al., 1995). Several other kinases are also able to phosphorylate these serine residues like ERK1/2, a downstream kinase of the MAPK pathway (Brady et al., 1998), p70 ribosomal S6 kinase-1 (Armstrong et al., 2001), cAMP-dependent protein kinase A (PKA) (Li et al., 2000), and PKC (Ballou et al., 2001).

Findings showing that inhibition of GSK3, siRNA-mediated depletion of GSK3β and absence of GSK3β in MEFs induced HIF-1α protein levels (Schnitzer et al., 2005; Flügel et al., 2007, 2012) were in line with the notion that GSK3 can phosphorylate at least HIF-1α. Indeed, GSK3β was found to directly phosphorylate HIF-1α in the ODDD and N-TAD (Sodhi et al., 2001; Flügel et al., 2007, 2012; Cassavaugh et al., 2011). The residues phosphorylated in HIF-1α by GSK3β were reported to be S551, T555, and S589 in one study (Flügel et al., 2007) whereas another study showed involvement of T498, S502, S505, T506, and S510 (Cassavaugh et al., 2011) (Figure 2). The difference between studies may have resulted from different oxygen concentrations (8% O_2_ compared to 2% O_2_) and the different cell types (HepG2 compared to SK-OV-3) used. Despite the different phosphorylation sites, both studies show that regulation of HIF-1α by GSK3β is independent of O_2_, hydroxylation, and VHL-mediated proteasomal degradation (Flügel et al., 2007, 2012; Cassavaugh et al., 2011). Thereby, phosphorylation of HIF-1α by GSK3β recruits the F-box and WD protein Fbw7 (also known as hCdc4 in yeast, hSel10 in *Caenorhabditis elegans*, or Ago in Drosophila) as the substrate-recognition component of a multi-subunit E3 ubiquitin ligase and forms together with SKP1 (S-phase kinase-associated protein 1), CUL1 (cullin 1), and RBX1 (RING box 1, also called ROC1 or HRT1) the so called SCF complex which then contributes to HIF-1α degradation (Cassavaugh et al., 2011; Flügel et al., 2012).

Similar to pVHL, Fbw7 is also a tumor suppressor; 6% from 1500 investigated human tumors showed mutations in the Fbw7 coding region. Strikingly, nearly half (43%) of these were missense mutations within the WD40 domain (Arg465 and Arg479), shared by all three alternatively spliced Fbw7 isoforms. In line, all three Fbw7 isoforms could target HIF-1α for proteasomal degradation and loss of the Fbw7 WD domain abolished GSK3β initiated HIF-1α degradation (Flügel et al., 2012).

Together, the findings showing that two different E3 substrate recognition proteins which both are tumor suppressors can contribute to HIFα degradation indicates the importance of the highly dynamic HIF system for carcinogenesis.

Ubiquitylation of proteins is reversible and the reversion is mediated by a family of deubiquitylating enzymes (DUBs). About 100 DUBs encoded by the human genome are supposed to counteract the action of around 600 E3 ligases (Nijman et al., 2005; Scheel and Hofmann, 2005). DUBs can be divided into five groups: ubiquitin-specific proteases (USPs), ubiquitin C-terminal hydrolases (UCHs), ovarian tumor proteases (OTUs), Josephins, and JAMMs. The USP, UCH, OUT, and Josephins are papain-like cysteine proteases, whereas the JAMM members are zinc metalloproteases (reviewed in Love et al., 2007).

Based on this, it appears plausible that the normoxia and pVHL-mediated ubiquitylation as well as the GSK3β and Fbw7-mediated ubiquitylation can be opposed by DUBs. Indeed, two VHL-interacting deubiquitylating enzymes, VDU1 (USP33) and VDU2 (USP20) were identified (Li et al., 2002a,b). However, by using *in vitro* pull down assays with GST-HIF-1α (amino acid 530–826) and co-immunoprecipitation experiments in COS-7 cells it was shown that only VDU2 but not VDU1 could interact with HIF-1α (Li et al., 2005). In addition, it was shown that VDU2 but not VDU1 can deubiquitylate HIF-1α and increase it's half-life (Li et al., 2005).

Experiments with GSK3β and Fbw7-deficient cells revealed that the GSK3β and Fbw7-dependent HIF-1α degradation can be antagonized by the ubiquitin specific protease 28 (USP28) (Flügel et al., 2012). In contrast to VDU2 which directly interacts with HIF-1α, USP28 forms a ternary complex with HIF-1α via its association with HIF-1α bound Fbw7 (Flügel et al., 2012).

Together, degradation of HIF-1α by the GSK3/Fbw7/USP28 system appears to be an additional mode to regulate HIF-1α function in response to various physiologic and non-physiologic signals affecting cell division, cell growth, differentiation, and apoptosis independent of the O_2_ tension.

While GSK3 provides a metabolic link to cell growth and differentiation, p38 MAP kinases link different stress stimuli, such as ultraviolet irradiation, heat shock, and osmotic shock with cell differentiation, apoptosis, and autophagy (Olson and Hallahan, 2004; Raman et al., 2007; Tormos et al., 2013; Sabio and Davis, 2014). Indeed, p38 was supposed to regulate HIF-1α stability during ischemic stress and in line, the p38 inhibitors SKF86002 and SB203580 decreased HIF-1 dependent gene expression (Sodhi et al., 2001). Further, treatment of the MiaPaca2 pancreatic cancer cell line with the p38 inhibitor SB203580 caused an increase in VHL-HIF-1α binding (Kwon et al., 2005) suggesting that p38 contributes to HIF-1α stabilization, though no half-life measurements were performed. Two members of the p38 MAPK family, p38α and p38γ, were then shown to possess the ability to phosphorylate HIF-1α (Sodhi et al., 2000). Altogether, this implies that p38 can contribute to HIF-1α stabilization, the phosphorylation by p38 occurred in the inhibitory domain (aa 576–785) (Sodhi et al., 2001) which has not yet been shown to be involved in VHL-dependent degradation. Moreover, the exact localization of the eight serine residues which could serve as putative p38 phosphorylation sites in the HIF-1α inhibitory domain as well as their contribution to HIFα degradation remains still to be determined (Figure 2).

Another kinase linking HIFα function with regulation of cell division is cyclin-dependent kinase 1 (CDK1). Although about 20 CDKs known to date can contribute to cell cycle control, CDK1 was found to be the only one essential for the cell cycle in all eukaryotic cells (Malumbres et al., 2009). CDK1 belongs to a highly conserved family of heterodimeric serine/threonine kinases which require a regulatory cyclin subunit for their activity. As such, the CDK1-cyclin B complex constitutes a serine/threonine protein kinase composed of the catalytic subunit CDK1 and its positive regulatory subunit cyclin B (B1 isoform) (Malumbres et al., 2009).

Activation of CDK1 promotes entry into the M phase of the cell cycle. This is achieved in the late G2 phase by phosphorylation mediated by the CDK activating kinase (CAK) phosphorylating T161 in its kinase-activation loop (Russo et al., 1996) as well as Cdc25C phosphatase mediated dephosphorylation of T14 and Y15 within CDK1. The inactive state of CDK1 throughout the S and G2 phases of the cell cycle is achieved by phosphorylation at two negative regulatory sites, T14 and Y15, by the CDK1 inhibitory protein kinases, Myt1 and Wee1 respectively (Watanabe et al., 2005) for review see (Malumbres, 2014, 2015).

A recent report showed that siRNA-mediated knockdown or Ro-3306-mediated inhibition of CDK1 reduced HIF-1α half-life whereas overexpression of CDK1 enhanced HIF-1α levels. *In vitro* kinase assays revealed that S668 in HIF-1α is the CDK1 target site (Figure 2). Accordingly, a construct of HIF-1α with a phospho-site mimicking mutation (S668E) was more stable under both normoxia and hypoxia. Moreover, phosphorylation of HIF-1α at S668 lead to an expression of HIF-1 target genes and promoted tumor angiogenesis, proliferation, and tumor growth (Warfel et al., 2013). Together, these findings underlie the importance of HIF-1α for the M-phase of the cell cycle since it can be stabilized by CDK1-mediated phosphorylation already under normoxia.

Genotoxic stress represents a burden under which cell cycle progression and cell cycle checkpoints need to be tightly controlled. A kinase participating in the response to genotoxic stresses is Polo-like kinase 3 (Plk3) (Barr et al., 2004). Plk3 is a member of a family consisting of four proteins (Plk1, Plk2, Plk3, and Plk4) not only involved in the stress response, but also strongly involved in tumorigenesis with an abnormal expression found in multiple tumors (Archambault and Glover, 2009; Degenhardt and Lampkin, 2010). The role of Plk3 in the development of tumors remains controversial. While one study showed a non-tumorigenic phenotype in Plk3 deficient mice (Myer et al., 2011), another study reported that mice deficient in Plk3 develop highly vascularized tumors in multiple organs suggesting a tumor-suppressing activity in particular via HIF driven angiogenesis (Yang et al., 2008). The latter finding is in line with the finding that Plk3 can regulate HIF-1α stability (Xu et al., 2010). Plk3 immunoprecipitation and pulldown analyses revealed interaction between HIF-1α and Plk3 which was able to phosphorylate S576 and S657 of HIF-1α (Xu et al., 2010) (Figure 2). Further, Plk3^−^/^−^ murine embryonic fibroblasts contained increased HIF-1α levels. In line with that, half-life measurements demonstrated that the half-life of wild-type HIF-1α was < 10 min, whereas the half-lives of the HIF-1α-S576A, HIF-1α-S657A, and HIF-1α-S576A/S657A mutants were about 37, 49, and 51 min, respectively (Xu et al., 2010). Together, these studies indicate that Plk3-mediated phosphorylation destabilizes HIF-1α.

In contrast to the above mentioned kinases, the knowledge about the involvement of the Jun N-terminal kinases (c-JNK) in regulating HIF-1α is quite limited and inconsistent. One study reported that c-JNK contributes to the activation of HIF-1α (Comerford et al., 2004) whereas other studies showed that HIF-1α is not phosphorylated by c-JNK (Richard et al., 1999; Sodhi et al., 2001).

#### Links between hypoxia and kinases in the regulation of HIFα stabilization

In addition to hormones or growth factors, hypoxia may also have an impact on the activity of certain kinases and thus activation of the hypoxia signal chain and a kinase pathway at the same time may lead to interference at the level of HIFα.

It has been shown that hypoxia is capable to induce GSK3β phosphorylation and thus its inactivation in different cell types such as PC-12 cells (Beitner-Johnson et al., 2001), HT1080 cells (Chen et al., 2001), and HepG2 cells (Mottet et al., 2003; Flügel et al., 2007) as well as *in vivo* (Roh et al., 2005). Further, early/acute hypoxia also enhanced PI3K/Akt activity, inhibited GSK3, and increased HIF-1α protein levels whereas prolonged/chronic hypoxia increased GSK3β activity which led to decreased HIF-1α protein levels in HepG2 cells (Mottet et al., 2003; Flügel et al., 2007). This indicates that hypoxia can also be a signal for the PI3K/Akt/GSK3 pathway and depending on the duration of hypoxia it is possible to induce a biphasic HIF-1α response. This would imply that GSK3β inhibition could reverse the negative effect of prolonged hypoxia on HIF-1α accumulation; however, these effects may be cell type specific since the hypoxia effects on GSK3β phosphorylation were not observed in other cell types including some different breast cancer cell lines (Blancher et al., 2000), PC-3 prostate cancer cells (Zhong et al., 2000), and 3T3 cells (Laughner et al., 2001).

GSK3 appears not to be the only kinase which may regulate HIFα stability by phosphorylation under normoxia and hypoxia. Recently it was found that the protein kinase ataxia-telangiectasia mutated (ATM) may be involved in the hypoxia-dependent modulation of HIF-1α function. Although ATM is best known for its role as an upstream activator of the DNA damage response due to DNA double-strand breaks (DSBs) (Shiloh and Ziv, 2013), it was described that ATM-deficient cells failed to accumulate HIF-1α under hypoxic conditions. In addition, ATM activity—but not protein—was found to be increased by about two-fold when NHFB cells were exposed to 0.2% oxygen; an increase in activity similar to that seen after ionizing irradiation. ATM was also able to phosphorylate HIF-1α at S696 in the ID and a HIF-1α S696A mutant was found to be less stable than wild-type HIF-1α under hypoxic conditions suggesting that S696 phosphorylation stabilizes HIF-1α (Cam et al., 2010) (Figure 2). However, not only stability but also activity of the HIF-1α S696A mutant was reduced with the consequence of reduced DNA-damage-inducible transcript 4 (DDIT4; also known as Dig2, HIF-1-responsive RTP801, REDD-1) expression (Shoshani et al., 2002). These features integrate the ATM DNA damage response pathway with the hypoxia signaling pathway.

In addition to hypoxia, reactive oxygen species (ROS) are also an important trigger of the DNA damage response and have been shown to be involved in the regulation of HIFα levels (Kietzmann and Gorlach, 2005; Gorlach and Kietzmann, 2007; Kietzmann, 2010). Although their major effects on HIFα stabilization are exerted via regulation of the proline hydroxylation- and VHL-dependent degradation pathway (Kietzmann and Gorlach, 2005; Gorlach and Kietzmann, 2007), also the PI3K/Akt and ERK1/2 pathway contributed to the ROS mediated HIFα regulation (Gorlach et al., 2001, 2003; Diebold et al., 2010). Recent studies in the roundworm *C. elegans* indicated that another kinase, namely AMP-activated protein kinase (AMPK) couples ROS and HIF-1α regulation in a direct manner. AMPK is a key sensor of the cellular energy status (Hardie et al., 2015) and considered to act downstream of reduced mitochondrial respiration. In their studies the authors demonstrated that mutations in the AMPK ortholog of *C. elegans* led to increased levels of HIF-1α indicating that AMPK is required for reducing HIF-1α. Further analyses revealed that AMPK regulates HIF-1α post-transcriptionally and by combining *in vitro* kinase assays with LC-MS analyses it was shown that AMPK phosphorylates S419 in *C. elegans* HIF-1α (Hwang et al., 2014) (Figure 2). Although, these data raise the possibility that AMPK down-regulates HIF-1α via direct phosphorylation, that study did not address to which extent this phosphorylation involves or requires VHL.

Although the *C. elegans* study also left open whether the direct regulation of HIFα is conserved among other species, it is known from studies with cancer cells that ROS-dependent HIF-1α activation requires AMPK (Jung et al., 2008). Interestingly and opposite to the regulation of HIF-1α by AMPK in *C. elegans* a recent study showed a link between AMPK function and HIF-1α regulation in the human hepatic cancer cell line Hep3B (Irigoyen et al., 1999; Chen et al., 2015). In these cells, the link between AMPK and HIF-1α appeared to be rather indirect involving histone deacetylase 5 (HDAC5) activity which can be phosphorylated by AMPK at S259 and S498. Since this phosphorylation of HDAC5 by AMPK promotes its shuttling from the nucleus to the cytosol (McKinsey et al., 2001) the authors examined whether cytosolic HDAC5 activity is involved in HIF-1α stabilization. They found that activation of AMPK by AICAR enhanced cytosolic presence of HDAC5 and levels of HIF-1α whereas the AMPK inhibitor compound C blocked HDAC5 nuclear export and HIF-1α accumulation (Irigoyen et al., 1999; Chen et al., 2015). Compound C, has also been shown to prevent hypoxia-dependent HIF-1α activation in DU145 cells (Lee et al., 2003; Hwang et al., 2004); however, this could be an independent effect since the inhibition of HIF-1α by compound C was also seen in AMPK^−∕−^ cells (Emerling et al., 2007). Together, it appears that AMPK can be involved in regulation of HIF-1α in a direct and indirect manner where the extent may be also depending on the species.

Altogether, these findings indicate that the HIFα system displays an enormous plasticity since its protein stabilization can be induced by hydroxylation and phosphorylation events either alone or in combination.

### Regulation of HIF α-subunit nuclear localization and transactivity by phosphorylation

Activation of multiple oncogenic pathways including growth factor signaling coupled with enhanced MAPK signaling is a common event in tumors (Raman et al., 2007). From the conventional MAP kinases the extracellular regulated kinases, ERK1 and ERK2 (p44/p42), c-Jun NH2-terminal kinase (JNK1/2/3), p38 MAPK (p38α/β/γ/δ) are known to be of importance for regulating cellular processes like proliferation, differentiation, development, stress responses, and apoptosis (Morrison and Davis, 2003; Olson and Hallahan, 2004; Coulombe and Meloche, 2007; Raman et al., 2007; Rincon and Davis, 2009; Gaestel, 2013; Serviddio et al., 2013; Tormos et al., 2013). Therefore, up-regulation of HIFα activity by MAPK signaling may play an essential role during tumor growth and metastasis.

**Figure 3:**
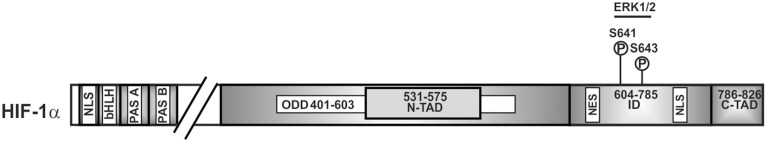
**Kinases involved in nuclear accumulation of HIF-1α**. Scheme of HIF-1α and its domain organization with specific amino acid residues phosphorylation of which affects nuclear translocation. ERK2, extracellular regulated kinase2; bHLH, basic helix loop helix domain; NLS, nuclear localization sequence; PAS, Per-ARNT-Sim domain; N-TAD, N-terminal transactivation domain; ID, inhibitory domain; C-TAD, C-terminal transactivation domain; numbers indicate the amino acid residue range of the respective domain.

To be able to act as transcription factor, stabilized HIFα proteins need to be translocated to the nucleus. This nuclear translocation was shown to be independent of ARNT and to be a dynamic process where nuclear import is commonly counterbalanced by nuclear export. Thus, the degree of nuclear HIFα accumulation depends on the relative nuclear import and export rates. In the case of HIFα, nuclear translocation was shown to involve its N-terminal and C-terminal NLS, respectively, as well as its interaction with importin 4 and 7 (Depping et al., 2015). The nuclear presence was then further shown to be regulated by ERK1 (p44)—and ERK2 (p42)-dependent phosphorylation. Thereby, mass spectroscopy with *in vitro* phosphorylated recombinant HIF-1α revealed that HIF-1α S641 and S643 (within the ID) served as p42/p44 MAPK targets (Mylonis et al., 2006; Triantafyllou et al., 2006) (Figure 3). Intriguingly, inhibition of these phosphorylation sites impaired HIF-1α nuclear accumulation and transcriptional activity by favoring nuclear export (Mylonis et al., 2006; Triantafyllou et al., 2006). This implies that ERK1/2 regulates rather the ability of HIFα to exit the nucleus rather than the import. Indeed, an atypical but CRM1 (exportin 1 or chromosome region maintenance)-dependent nuclear export signal (NES) (within aa 616–658 in HIF-1α) (Mylonis et al., 2008) was found to be phosphorylation-sensitive. Phosphorylation of S641 and S643 within the NES by ERK1/2 inhibited interaction between HIF-1α and the exporting CRM1 and facilitated nuclear accumulation.

In line with the nuclear accumulation are findings reporting that enhanced transcriptional activity of both HIF-1α and HIF-2α can be observed after direct phosphorylation of the HIFα isoforms by ERK1/2 *in vitro* and *in vivo* (Richard et al., 1999; Conrad et al., 2000; Minet et al., 2000; Sang et al., 2003; Mylonis et al., 2008). In line, the MEK1 inhibitor PD98059 and MKK inhibitor U0126 decreased HIF-target gene expression (Hur et al., 2001; Sodhi et al., 2001; Comerford et al., 2004; Dimova et al., 2005; Kaluz et al., 2006).

While the C-TAD of HIF-1α and HIF-2α is important for recruitment of the coactivator CBP/p300, phosphorylation sites within the C-TAD (Minet et al., 2000; Sodhi et al., 2001; Lee et al., 2002) and within the ID (Sodhi et al., 2001; Lee et al., 2002; Sang et al., 2003) of HIFα may also contribute to induction of transactivity. Indeed, the first functionally relevant phosphorylation sites were reported to be T796 in HIF-1α and T844 in HIF-2α (Gradin et al., 2002). Although the kinases phosphorylating these sites were not defined, phosphorylation of these residues increased interaction between the HIFα-C-TAD and CBP/p300. Moreover, it was shown that the MEK1 inhibitor PD98059 affected the transactivity of CBP/p300 and that ERK1 could also phosphorylate the transactivation domain of p300 (aa 1751–2414) which subsequently facilitated interaction between the HIF-1α C-TAD and p300 (Sang et al., 2003). Together with the finding that phosphorylated HIF-1α is the major form binding to ARNT (Suzuki et al., 2001), it appears plausible that HIF-1 transcriptional activity increases in response to induction of the MAPK pathway.

All together, these reports indicate that direct phosphorylation of HIF-1α and HIF-2α by ERK1/2 can affect their nuclear localization and transactivity.

#### Links between hypoxia and kinases in the regulation of HIF-1α transactivity

**Figure 4:**
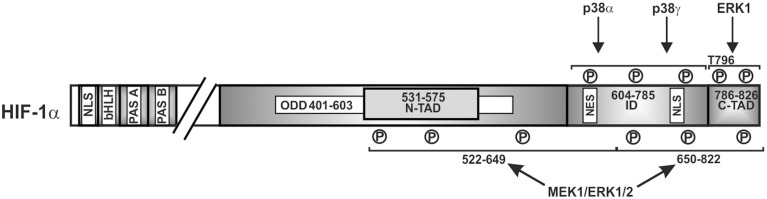
**Kinases involved in regulating HIF-1α transactivity**. Scheme of HIF-1α and its domains in which phosphorylation has been shown to affect transactivity. P, represents a phosphorylated amino acid, no specific single residues sites have been mapped. ERK1/2, extracellular regulated kinase1/2; bHLH, basic helix loop helix domain; NLS, nuclear localization sequence; PAS, Per-ARNT-Sim domain; N-TAD, N-terminal transactivation domain; ID, inhibitory domain; C-TAD, C-terminal transactivation domain; numbers indicate the amino acid residue range of the respective domain.

A number of findings have indicated that ERK1/2 can also serve as additional transmitter of the hypoxic signal since hypoxia has been shown to moderately activate ERK1/2 in different cell lines (Salceda et al., 1997; Conrad et al., 1999; Minet et al., 2000). Thereby cell type specific variations may appear as shown for HMEC-1 cells where involvement of ERK1 but not ERK2 in hypoxia-mediated HIF-1 transactivation was reported (Minet et al., 2000). In addition, by using PD98059 and by employing a mammalian two-hybrid assay, it was shown that the ERK pathway is also involved in hypoxia-dependent HIF-1α transactivation (Lee et al., 2002; Sang et al., 2003). By contrast, ERK1/2 activity was not increased in hypoxic growth-arrested Chinese hamster fibroblast CCL39 cells (Richard et al., 1999) implying that an activation of either ERK1 or ERK2 in response to hypoxia as well as their involvement in HIFα regulation may be cell type specific. Accordingly, the MEK1 inhibitor PD98059 suppressed hypoxia-mediated HIF-1α transcriptional activity in Hep3B and HMEC-1 cells (Salceda et al., 1997; Minet et al., 2000) whereas the same inhibitor was ineffective in fibroblasts exposed to hypoxia (Agani and Semenza, 1998). However, in all these studies direct mapping of the involved residues within HIFα proteins were not performed; thus only an approximate localization can be given (Figure 4).

In addition to ERK1/2, protein kinase CK2 (formerly known as casein kinase II) has important functions in the regulation of various cellular processes (Niefind et al., 2009; St-Denis and Litchfield, 2009; Montenarh, 2010). CK2 was shown to affect HIF-1α transcriptional activity (Mottet et al., 2005; Hubert et al., 2006); however, the exact mechanisms and CK2 phosphorylation sites in HIF-1α were not determined; likely CK2-mediated HIF-1α phosphorylation prevents recruitment of cofactors like CBP/p300 or stimulates HIF-1α degradation in an indirect manner (see below).

Together, these findings indicate an interrelation between hypoxia, ERK1/2, and CK2 signaling pathways in particular for the regulation of HIF-1α transactivity.

## Kinases regulating HIFα abundance in an indirect manner

In addition to being a direct substrate for kinases, HIFα appears to be regulated via phosphorylation of HIFα regulating proteins in an indirect manner.

The protein kinase A (PKA) is among the best characterized kinases and was suggested to be involved in HIF-1α phosphorylation under intermittent hypoxia in EAhy926 endothelial cells (Toffoli et al., 2007). However, from that study it remained open whether or not HIFα proteins can be direct substrates for that kinase since no functional phosphorylation site(s) was identified yet.

**Figure 5:**
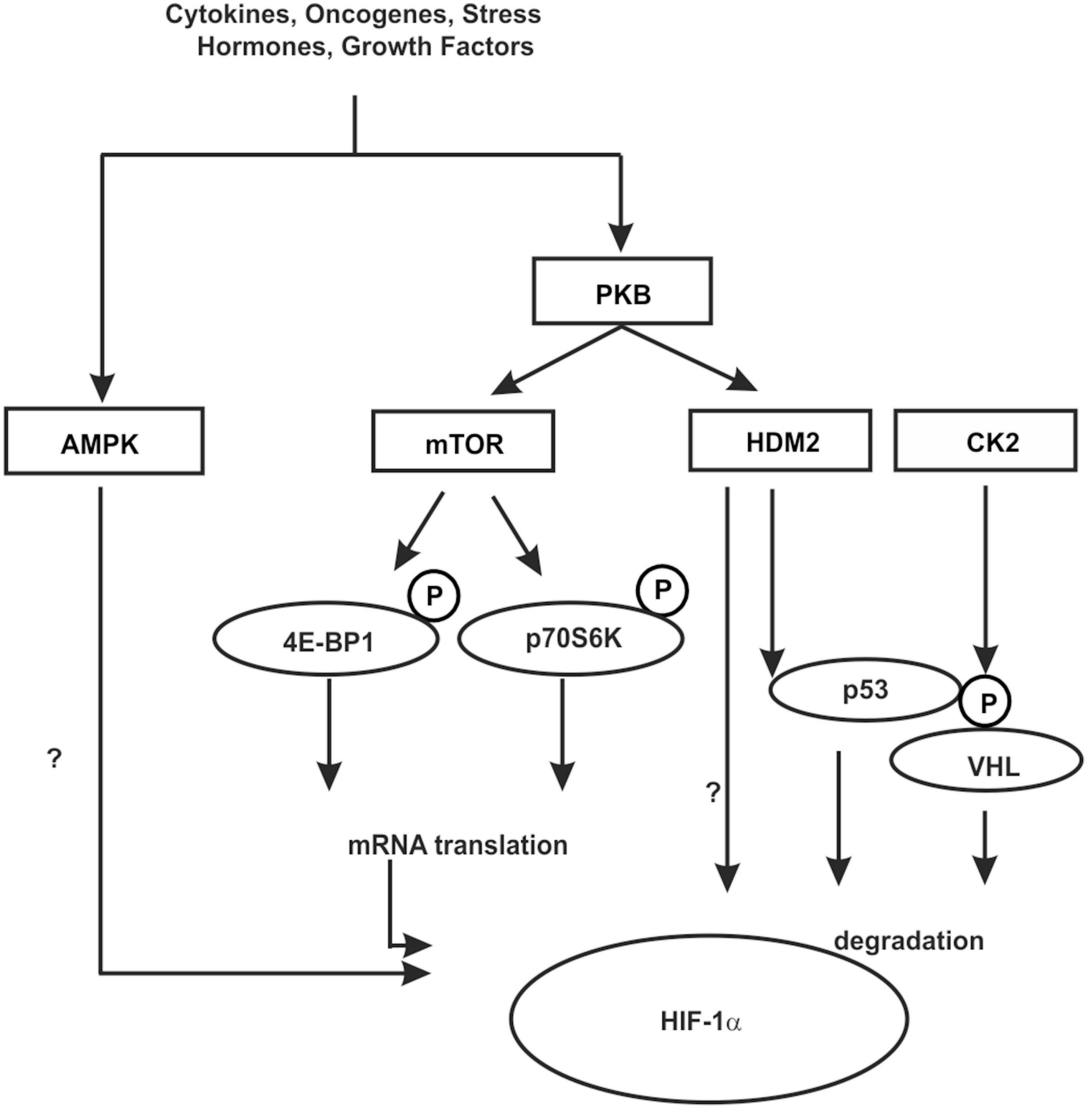
**Kinases contributing to HIF-1α regulation in an indirect manner**. In response to various hormones, growth factors, cytokines, oncogenes, and stress phosphorylation events can be initiated which contribute to the regulation of HIF-1α in an indirect manner. These can influence HIF-1α mRNA translation, the interaction with cofactors or components of the protein degradation machinery like HDM2 and VHL. Some kinases may act in both ways, however, the knowledge about the exact mechanisms is limited. See text for more details.

As mentioned above, protein kinase CK2, a constitutive serine/threonine kinase which interestingly shows high CK2 activity in most human cancers can indirectly contribute to HIF-1α degradation. Thereby, CK2 phosphorylates S33, S38, and S43 within VHL. Mutation of the CK2 sites within VHL or inhibition of VHL phosphorylation with CK2 inhibitors increased VHL protein half-life and promoted degradation of HIF-1α (Ampofo et al., 2010). At the same time inhibited CK2 could also sequester p53 and reduce the transcriptional activity of p53. Together, this indicates that the indirect action of CK2 on HIF-1α and p53 can contribute to the survival of tumor cells (Figure 5).

One of the best known kinase pathways affecting HIFα in an indirect manner by regulating HIFα protein synthesis involves the mammalian target of rapamycin (mTOR). The mTOR is a serine/threonine protein kinase [also known as FK506 binding protein 12-rapamycin associated protein 1 (FRAP1)] (Brown et al., 1994; Moore et al., 1996) that apart from cell growth, cell proliferation, cell motility, cell survival, and transcription contributes to the regulation of protein synthesis in response to nutrients, hormones, growth factors, cytokines, and stress (for review see Hay and Sonenberg, 2004; Beevers et al., 2006; Dunlop and Tee, 2009). Thereby mTOR regulates translation primarily via phosphorylation of eukaryotic initiation factor 4E-binding protein 1 (4E-BP1) and ribosomal S6 kinase (S6K) (reviewed by Hay and Sonenberg, 2004; Inoki et al., 2005). By binding to translation initiation factor 4E (eIF4E) 4E-BP1 prevents interaction of eIF4E with other members of the translation initiation complex and inhibits ribosomal complex formation at the 5′-cap mRNAs. The phosphorylation of 4E-BP1 by mTOR results in its dissociation from eIF4E and in activation of mRNA translation (reviewed by Hay and Sonenberg, 2004; Inoki et al., 2005). In addition, phosphorylation of ribosomal S6K promotes translation of mRNAs containing a terminal oligopyrimidine tract (5′TOP) in their 5′-UTR (Figure 5).

Two major multiprotein complexes can be distinguished in which mTOR contributes to signaling; (i) the rapamycin-sensitive mTOR complex 1 (mTORC1) and (ii) the rapamycin-insensitive mTOR complex 2 (mTORC2) (Wullschleger et al., 2006). Several excellent reviews discussing in detail the composition of the TOR complexes and the impact of the participating proteins for signaling are available (see Hay and Sonenberg, 2004; Dunlop and Tee, 2009) and therefore we limit ourselves to the issue of HIFα regulation. While mTORC1 appears to be involved in nutrient/energy/redox sensing, mTORC2 seems to be mainly regulated by insulin, growth factors, serum and nutrients (Kim et al., 2003; Sarbassov et al., 2004; Frias et al., 2006).

The participation of mTOR in the regulation of HIF-1α protein translation was first shown in a study with breast cancer cells where stimulation with heregulin and HER2 signaling increased the rate of HIF-1α synthesis in a rapamycin-dependent manner (Laughner et al., 2001). Other studies in HUVEC and HeLa cells (Kim et al., 2009) supported that view and also showed that not only HIF-1α but also HIF-2α was found to be regulated by mTOR signaling, though HIF-1α expression seems to be regulatable by TORC1 and TORC2 whereas HIF-2α expression is primarily dependent on TORC2 (Toschi et al., 2008).

Hypoxia has been reported to inhibit mTOR (Arsham et al., 2003) via induction of the hypoxia-responsive gene DDIT4 (Dig2/RTP801/REDD1) and subsequent formation of a complex consisting of the tuberous sclerosis tumor suppressor proteins TSC1 (hamartin) and TSC2 (tuberin) (Brugarolas et al., 2004). The TSC1/TSC2 complex inhibits primarily mTORC1 signaling; destruction/inhibition of the TSC1/2 complex due to growth factors leads to activation of mTORC1 signaling (Hay and Sonenberg, 2004). For removal of TSC2 different kinase pathways, including PI3K/AKT, and ERK1/2 appear to be important (Hay and Sonenberg, 2004). Once phosphorylated, TSC2 can be captured by 14-3-3 proteins, thus leaving the complex with TSC1 and rendering mTORC1 active (Li et al., 2003).

Reciprocally, the hypoxia mediated inhibition of mTORC1 signaling (Brugarolas et al., 2004) appeared to be the result of a dissociation of TSC2 from the growth factor stimulated TSC2/14-3-3 complex. Thereby, hypoxic induction of DDIT4 seemed to be critical. Due to the ability of DDIT4 to bind 14-3-3 proteins this resulted in a release of TSC2 with formation of TSC1/2 complexes which subsequently inhibited of mTORC1 (DeYoung et al., 2008).

Thus, DDIT4 and TSC1/TSC2 formation could decrease mTOR activity and would reduce HIF-1α translation under hypoxia. However, under hypoxia when the cellular protein translation is generally suppressed, HIF-1α is still translated. This occurs likely by a mechanism involving the 5′-UTR of the HIF-1α mRNA which contains a terminal oligopyrimidine tract that enables HIF-1α translation even when mTOR is inhibited (Laughner et al., 2001; Thomas et al., 2006). Like with mTOR, ATR (for ataxia telangiectasia and Rad3 related kinase) appeared also to regulate HIF-1α translation in a region located within the HIF-1α ORF (Fallone et al., 2013).

The involvement of mTOR in HIF-1α translation was challenged in studies showing that rapamycin decreased hypoxia-induced HIF-1α stability at the ODD in PC-3 cells (Hudson et al., 2002; Dayan et al., 2009). Further, mTORC1 appeared to act also directly on HIF-1α since an mTOR signaling motif (FVMVL) modulating recruitment of CBP/p300 was found immediately C-terminal of the PAS-A domain in HIF-1α (Land and Tee, 2007). Thus, although mTOR signaling appears to affect HIFα abundance in a more indirect manner, it appears that also direct interactions are possible which may depend on the stimulus.

Growth factor stimulation and hence kinase signaling is not only important for mTOR signaling but also for crosstalk between the HIF-1α and the p53 network (Fukuda et al., 2002; Bardos et al., 2004). The murine double minute-2 (mdm2) and its human ortholog HDM2 protein are negative regulators of the p53 tumor suppressor protein (for review see Eischen and Lozano, 2009; Kruiswijk et al., 2015). In addition to p53, HDM2, which is a direct target of PKB/Akt (Ashcroft et al., 2002), was shown to regulate HIF-1α expression in response to IGF-1 in p53-null mouse embryo fibroblasts (p53^−∕−^ MEFs) (Bardos et al., 2004). Moreover, this appeared to involve protein synthesis and HDM2 phosphorylation at S166 by PKB/Akt (Bardos et al., 2004) suggesting that the PKB/Akt pathway also affects HIF-1α synthesis via HDM2 in a p53 independent manner (Figure 5).

Altogether, kinases regulating HIFα synthesis or degradation by acting on critical regulators of these processes are important mediators which interlink growth factor controlled pathways with hypoxia signaling.

## Exploiting kinases as upstream regulators of HIF-1α in cancer therapy

An impaired regulation of kinase signaling is associated with a number of systemic diseases including cardiovascular diseases, pulmonary diseases, Alzheimer's disease, type 2 diabetes mellitus, and last but not least cancer. In particular, intermittent hypoxia in pre-malignant lesions and HIF-1α were proposed to contribute to the reprogramming of metabolism toward permanent conversion of glucose to lactate even in aerobic conditions (Gatenby and Gillies, 2004) known as the “Warburg effect,” mitochondrial suppression as well as to acidosis. This provides a growth advantage, and an altered response to growth factors which are major actors on kinase signaling pathways. Thus, the interconnection of kinase signaling pathways and hypoxia signaling, i.e., HIFα regulation, is of high therapeutic interest. This is most obvious in cancer therapy where different kinase inhibitors are in clinical use and where severe hypoxic tumors are more resistant to chemotherapy and radiation. Interestingly the most successful kinase inhibitors currently used in cancer therapy are tyrosine kinase inhibitors like imatinib, gefitinib, and erlotinib. Tyrosine kinases are often found to act as receptors for hormones and growth factors and therefore they appear often to have an effect also on HIF-1α which is either direct or indirect (Figure 5). In addition to the tyrosine kinase inhibitors, other small molecules with the potential to act on MAPK, mTOR, or Akt pathways are under heavy investigation. Interestingly, the inhibitor of pyruvate dehydrogenase kinase II, dichloroacetate, has been shown to reactivate mitochondria via inhibition of HIF1α involving a PHD-dependent mechanism and a PHD-independent mechanism, involving activation of p53 and GSK3β (Sutendra et al., 2013).

However, these inhibitors are often not entirely specific but rather selective which explains their variety of actions as well as their effectiveness also in other disorders, including immunological, neurological, metabolic, and infectious diseases. Although this is already an advantage, it is difficult to predict to which extent kinase inhibitors could be made selective or even specific to target the HIF pathway. This is not only complicated by the fact that the respective kinase having a dominant role in HIFα regulation needs to have a role in the particular tumor entity. Thus, significant challenges remain. In addition to quick evolvement of tumors resistant to kinases inhibitors, appropriate multi-targeted inhibitors or combinations appear currently to be of advance in clinical therapy. Further, more understanding of the kinase inhibitor specificities toward HIF-1α, metabolic and toxic side effects would be needed to optimize cancer therapy.

## Conclusion

Detailed knowledge about the kinase pathways and their effect on HIFα regulation is essential to optimize and to develop highly efficient cancer therapies. It is now especially necessary to gather more knowledge about the involvement of kinase pathways for the regulation of HIF-2α and HIF-3α since most of the data so far, with respect to kinases and HIFα regulation, have been gained from studies on HIF-1α. Given that certain aspects between HIF-1α and HIF-2α as well as the occurrence of several splice variants of HIF-3α point to more different roles of each HIFα protein in a number of processes, it is obvious that this knowledge would be beneficial for therapeutic purposes.

Overall, the HIFα system appears to be a central integrator of various signals coming from different pathways. Thereby it displays an enormous plasticity being regulated by a number of post-translational modifications, among them phosphorylation, either alone or in combination.

## Author contributions

TK, DM, and ED, have written the manuscript and generated the data and figures.

### Conflict of interest statement

The authors declare that the research was conducted in the absence of any commercial or financial relationships that could be construed as a potential conflict of interest.

## References

[B1] Agani F. Semenza G. L. (1998). Mersalyl is a novel inducer of vascular endothelial growth factor gene expression and hypoxia-inducible factor 1 activity. Mol. Pharmacol. 54, 749–754. 9804609 10.1124/mol.54.5.749

[B2] Ampofo E. Kietzmann T. Zimmer A. Jakupovic M. Montenarh M. Gotz C. (2010). Phosphorylation of the von Hippel-Lindau protein (VHL) by protein kinase CK2 reduces its protein stability and affects p53 and HIF-1α mediated transcription. Int. J. Biochem. Cell Biol. 42, 1729–1735. 10.1016/j.biocel.2010.07.008 20637892

[B3] Archambault V. Glover D. M. (2009). Polo-like kinases: conservation and divergence in their functions and regulation. Nat. Rev. Mol. Cell Biol. 10, 265–275. 10.1038/nrm2653 19305416

[B4] Armstrong J. L. Bonavaud S. M. Toole B. J. Yeaman S. J. (2001). Regulation of glycogen synthesis by amino acids in cultured human muscle cells. J. Biol. Chem. 276, 952–956. 10.1074/jbc.M004812200 11013237

[B5] Arsham A. M. Howell J. J. Simon M. C. (2003). A novel hypoxia-inducible factor-independent hypoxic response regulating mammalian target of rapamycin and its targets. J. Biol. Chem. 278, 29655–29660. 10.1074/jbc.M212770200 12777372

[B6] Ashcroft M. Ludwig R. L. Woods D. B. Copeland T. D. Weber H. O. MacRae E. J.. (2002). Phosphorylation of HDM2 by Akt. Oncogene 21, 1955–1962. 10.1038/sj.onc.1205276 11960368

[B7] Ballou L. M. Tian P. Y. Lin H. Y. Jiang Y. P. Lin R. Z. (2001). Dual regulation of glycogen synthase kinase-3β by the α1A-adrenergic receptor. J. Biol. Chem. 276, 40910–40916. 10.1074/jbc.M103480200 11533051

[B8] Bardos J. I. Chau N. M. Ashcroft M. (2004). Growth factor-mediated induction of HDM2 positively regulates hypoxia-inducible factor 1α expression. Mol. Cell. Biol. 24, 2905–2914. 10.1128/MCB.24.7.2905-2914.2004 15024078 PMC371114

[B9] Barr F. A. Sillje H. H. Nigg E. A. (2004). Polo-like kinases and the orchestration of cell division. Nat. Rev. Mol. Cell Biol. 5, 429–440. 10.1038/nrm1401 15173822

[B10] Beevers C. S. Li F. Liu L. Huang S. (2006). Curcumin inhibits the mammalian target of rapamycin-mediated signaling pathways in cancer cells. Int. J. Cancer 119, 757–764. 10.1002/ijc.21932 16550606

[B11] Beitner-Johnson D. Rust R. T. Hsieh T. C. Millhorn D. E. (2001). Hypoxia activates Akt and induces phosphorylation of GSK-3 in PC12 cells. Cell. Signal. 13, 23–27. 10.1016/S0898-6568(00)00128-5 11257444

[B12] Berra E. Benizri E. Ginouves A. Volmat V. Roux D. Pouyssegur J. (2003). HIF prolyl-hydroxylase 2 is the key oxygen sensor setting low steady-state levels of HIF-1α in normoxia. EMBO J. 22, 4082–4090. 10.1093/emboj/cdg392 12912907 PMC175782

[B13] Blancher C. Moore J. W. Talks K. L. Houlbrook S. Harris A. L. (2000). Relationship of hypoxia-inducible factor (HIF)-1 α and HIF-2 α expression to vascular endothelial growth factor induction and hypoxia survival in human breast cancer cell lines. Cancer Res. 60, 7106–7113. 11156418

[B14] Brady M. J. Bourbonais F. J. Saltiel A. R. (1998). The activation of glycogen synthase by insulin switches from kinase inhibition to phosphatase activation during adipogenesis in 3T3-L1 cells. J. Biol. Chem. 273, 14063–14066. 10.1074/jbc.273.23.14063 9603900

[B15] Brown E. J. Albers M. W. Shin T. B. Ichikawa K. Keith C. T. Lane W. S.. (1994). A mammalian protein targeted by G1-arresting rapamycin-receptor complex. Nature 369, 756–758. 10.1038/369756a0 8008069

[B16] Brugarolas J. Lei K. Hurley R. L. Manning B. D. Reiling J. H. Hafen E.. (2004). Regulation of mTOR function in response to hypoxia by REDD1 and the TSC1/TSC2 tumor suppressor complex. Genes Dev. 18, 2893–2904. 10.1101/gad.1256804 15545625 PMC534650

[B17] Cam H. Easton J. B. High A. Houghton P. J. (2010). mTORC1 signaling under hypoxic conditions is controlled by ATM-dependent phosphorylation of HIF-1α. Mol. Cell 40, 509–520. 10.1016/j.molcel.2010.10.030 21095582 PMC3004768

[B18] Cassavaugh J. M. Hale S. A. Wellman T. L. Howe A. K. Wong C. Lounsbury K. M. (2011). Negative regulation of HIF-1α by an FBW7-mediated degradation pathway during hypoxia. J. Cell. Biochem. 112, 3882–3890. 10.1002/jcb.23321 21964756 PMC3202039

[B19] Chen E. Y. Mazure N. M. Cooper J. A. Giaccia A. J. (2001). Hypoxia activates a platelet-derived growth factor receptor/phosphatidylinositol 3-kinase/Akt pathway that results in glycogen synthase kinase-3 inactivation. Cancer Res. 61, 2429–2433. 11289110

[B20] Chen S. Yin C. Lao T. Liang D. He D. Wang C.. (2015). AMPK-HDAC5 pathway facilitates nuclear accumulation of HIF-1a and functional activation of HIF-1 by deacetylating Hsp70 in the cytosol. Cell Cycle 14, 2520–2536. 10.1080/15384101.2015.1055426 26061431 PMC4614078

[B21] Cohen P. Frame S. (2001). The renaissance of GSK3. Nat. Rev. 2, 769–776. 10.1038/35096075 11584304

[B22] Comerford K. M. Cummins E. P. Taylor C. T. (2004). c-Jun NH2-terminal kinase activation contributes to hypoxia-inducible factor 1α-dependent P-glycoprotein expression in hypoxia. Cancer Res. 64, 9057–9061. 10.1158/0008-5472.CAN-04-1919 15604272

[B23] Conrad P. W. Freeman T. L. Beitner-Johnson D. Millhorn D. E. (1999). EPAS1 trans-activation during hypoxia requires p42/p44 MAPK. J. Biol. Chem. 274, 33709–33713. 10.1074/jbc.274.47.33709 10559262

[B24] Conrad P. W. Millhorn D. E. Beitner-Johnson D. (2000). Hypoxia differentially regulates the mitogen- and stress-activated protein kinases. Role of Ca2+/CaM in the activation of MAPK and p38 γ. Adv. Exp. Med. Biol. 475, 293–302. 10.1007/0-306-46825-5_28 10849670

[B25] Coulombe P. Meloche S. (2007). Atypical mitogen-activated protein kinases: structure, regulation and functions. Biochim. Biophys. Acta 1773, 1376–1387. 10.1016/j.bbamcr.2006.11.001 17161475

[B26] Cross D. A. Alessi D. R. Cohen P. Andjelkovich M. Hemmings B. A. (1995). Inhibition of glycogen synthase kinase-3 by insulin mediated by protein kinase B. Nature 378, 785–789. 10.1038/378785a0 8524413

[B27] Dayan F. Bilton R. L. Laferriere J. Trottier E. Roux D. Pouyssegur J.. (2009). Activation of HIF-1α in exponentially growing cells via hypoxic stimulation is independent of the Akt/mTOR pathway. J. Cell. Physiol. 218, 167–174. 10.1002/jcp.21584 18781596

[B28] Degenhardt Y. Lampkin T. (2010). Targeting Polo-like kinase in cancer therapy. Clin. Cancer Res. 16, 384–389. 10.1158/1078-0432.CCR-09-1380 20068088

[B29] Depping R. Jelkmann W. Kosyna F. K. (2015). Nuclear-cytoplasmatic shuttling of proteins in control of cellular oxygen sensing. J. Mol. Med. (Berl.) 93, 599–608. 10.1007/s00109-015-1276-0 25809665

[B30] DeYoung M. P. Horak P. Sofer A. Sgroi D. Ellisen L. W. (2008). Hypoxia regulates TSC1/2-mTOR signaling and tumor suppression through REDD1-mediated 14-3-3 shuttling. Genes Dev. 22, 239–251. 10.1101/gad.1617608 18198340 PMC2192757

[B31] Diebold I. Flügel D. Becht S. Belaiba R. S. Bonello S. Hess J.. (2010). The hypoxia-inducible factor-2α is stabilized by oxidative stress involving NOX4. Antioxid. Redox Signal. 13, 425–436. 10.1089/ars.2009.3014 20039838

[B32] Dimova E. Y. Michiels C. Kietzmann T. (2009). Kinases as upstream regulators of the HIF system: their emerging potential as anti-cancer drug targets. Curr. Pharm. Des. 15, 3867–3877. 10.2174/138161209789649358 19671044

[B33] Dimova E. Y. Moller U. Herzig S. Fink T. Zachar V. Ebbesen P.. (2005). Transcriptional regulation of plasminogen activator inhibitor-1 expression by insulin-like growth factor-1 via MAP kinases and hypoxia-inducible factor-1 in HepG2 cells. Thromb. Haemost. 93, 1176–1184. 10.1160/th04-11-0761 15968405

[B34] Duan C. (2015). Hypoxia-inducible factor 3 biology: complexities and emerging themes. Am. J. Physiol. Cell. Physiol. [Epub ahead of print]. 10.1152/ajpcell.00315.2015. 26561641

[B35] Dunlop E. A. Tee A. R. (2009). Mammalian target of rapamycin complex 1: signalling inputs, substrates and feedback mechanisms. Cell. Signal. 21, 827–835. 10.1016/j.cellsig.2009.01.012 19166929

[B36] Eischen C. M. Lozano G. (2009). p53 and MDM2: antagonists or partners in crime? Cancer Cell. 15, 161–162. 10.1016/j.ccr.2009.02.004 19249672

[B37] Ema M. Taya S. Yokotani N. Sogawa K. Matsuda Y. Fujii-Kuriyama Y. (1997). A novel bHLH-PAS factor with close sequence similarity to hypoxia-inducible factor 1α regulates the VEGF expression and is potentially involved in lung and vascular development. Proc. Natl. Acad. Sci. U.S.A. 94, 4273–4278. 10.1073/pnas.94.9.4273 9113979 PMC20712

[B38] Emerling B. M. Viollet B. Tormos K. V. Chandel N. S. (2007). Compound C inhibits hypoxic activation of HIF-1 independent of AMPK. FEBS Lett. 581, 5727–5731. 10.1016/j.febslet.2007.11.038 18036344 PMC2169511

[B39] Fallone F. Britton S. Nieto L. Salles B. Muller C. (2013). ATR controls cellular adaptation to hypoxia through positive regulation of hypoxia-inducible factor 1 (HIF-1) expression. Oncogene 32, 4387–4396. 10.1038/onc.2012.462 23085754

[B40] Flamme I. Frohlich T. von Reutern M. Kappel A. Damert A. Risau W. (1997). HRF, a putative basic helix-loop-helix-PAS-domain transcription factor is closely related to hypoxia-inducible factor-1 α and developmentally expressed in blood vessels. Mech. Dev. 63, 51–60. 10.1016/S0925-4773(97)00674-6 9178256

[B41] Flügel D. Gorlach A. Kietzmann T. (2012). GSK-3β regulates cell growth, migration, and angiogenesis via Fbw7 and USP28-dependent degradation of HIF-1α. Blood 119, 1292–1301. 10.1182/blood-2011-08-375014 22144179 PMC3352078

[B42] Flügel D. Gorlach A. Michiels C. Kietzmann T. (2007). Glycogen synthase kinase 3 phosphorylates hypoxia-inducible factor 1α and mediates its destabilization in a VHL-independent manner. Mol. Cell. Biol. 27, 3253–3265. 10.1128/MCB.00015-07 17325032 PMC1899978

[B43] Force T. Woodgett J. R. (2009). Unique and overlapping functions of GSK-3 isoforms in cell differentiation and proliferation and cardiovascular development. J. Biol. Chem. 284, 9643–9647. 10.1074/jbc.R800077200 19064989 PMC2665084

[B44] Frias M. A. Thoreen C. C. Jaffe J. D. Schroder W. Sculley T. Carr S. A.. (2006). mSin1 is necessary for Akt/PKB phosphorylation, and its isoforms define three distinct mTORC2s. Curr. Biol. 16, 1865–1870. 10.1016/j.cub.2006.08.001 16919458

[B45] Fukuda R. Hirota K. Fan F. Jung Y. D. Ellis L. M. Semenza G. L. (2002). Insulin-like growth factor 1 induces hypoxia-inducible factor 1-mediated vascular endothelial growth factor expression, which is dependent on MAP kinase and phosphatidylinositol 3-kinase signaling in colon cancer cells. J. Biol. Chem. 277, 38205–38211. 10.1074/jbc.M203781200 12149254

[B46] Gaestel M. (2013). What goes up must come down: molecular basis of MAPKAP kinase 2/3-dependent regulation of the inflammatory response and its inhibition. Biol. Chem. 394, 1301–1315. 10.1515/hsz-2013-0197 23832958

[B47] Gatenby R. A. Gillies R. J. (2004). Why do cancers have high aerobic glycolysis? Nat. Rev. Cancer 4, 891–899. 10.1038/nrc1478 15516961

[B48] Gorlach A. (2009). Regulation of HIF-1α at the transcriptional level. Curr. Pharm. Des. 15, 3844–3852. 10.2174/138161209789649420 19671046

[B49] Gorlach A. Berchner-Pfannschmidt U. Wotzlaw C. Cool R. H. Fandrey J. Acker H.. (2003). Reactive oxygen species modulate HIF-1 mediated PAI-1 expression: Involvement of the GTPase Rac1. Thromb. Haemost. 89, 927–935. 10.1267/THRO03050926 12719791

[B50] Gorlach A. Diebold I. Schini-Kerth V. B. Berchner-Pfannschmidt U. Roth U. Brandes R. P.. (2001). Thrombin activates the hypoxia-inducible factor-1 signaling pathway in vascular smooth muscle cells: Role of the p22(phox)-containing NADPH oxidase. Circ. Res. 89, 47–54. 10.1161/hh1301.092678 11440977

[B51] Gorlach A. Kietzmann T. (2007). Superoxide and derived reactive oxygen species in the regulation of hypoxia-inducible factors. Methods Enzymol. 435, 421–446. 10.1016/S0076-6879(07)35022-2 17998067

[B52] Gradin K. Takasaki C. Fujii-Kuriyama Y. Sogawa K. (2002). The transcriptional activation function of the HIF-like factor requires phosphorylation at a conserved threonine. J. Biol. Chem. 277, 23508–23514. 10.1074/jbc.M201307200 11983697

[B53] Grimes C. A. Jope R. S. (2001). The multifaceted roles of glycogen synthase kinase 3β in cellular signaling. Prog. Neurobiol. 65, 391–426. 10.1016/S0301-0082(01)00011-9 11527574

[B54] Gross J. Rheinlander C. Fuchs J. Mazurek B. Machulik A. Andreeva N.. (2003). Expression of hypoxia-inducible factor-1 in the cochlea of newborn rats. Hear. Res. 183, 73–83. 10.1016/S0378-5955(03)00222-3 13679140

[B55] Hara S. Hamada J. Kobayashi C. Kondo Y. Imura N. (2001). Expression and characterization of hypoxia-inducible factor (HIF)-3α in human kidney: suppression of HIF-mediated gene expression by HIF-3α. Biochem. Biophys. Res. Commun. 287, 808–813. 10.1006/bbrc.2001.5659 11573933

[B56] Hardie D. G. Schaffer B. E. Brunet A. (2015). AMPK: an energy-sensing pathway with multiple inputs and outputs. Trends Cell Biol. [Epub ahead of print]. 10.1016/j.tcb.2015.10.013. 26616193 PMC5881568

[B57] Hay N. Sonenberg N. (2004). Upstream and downstream of mTOR. Genes Dev. 18, 1926–1945. 10.1101/gad.1212704 15314020

[B58] Heikkila M. Pasanen A. Kivirikko K. I. Myllyharju J. (2011). Roles of the human hypoxia-inducible factor (HIF)-3α variants in the hypoxia response. Cell Mol. Life Sci. 68, 3885–3901. 10.1007/s00018-011-0679-5 21479871 PMC11114783

[B59] Hewitson K. S. McNeill L. A. Riordan M. V. Tian Y. M. Bullock A. N. Welford R. W.. (2002). Hypoxia-inducible factor (HIF) asparagine hydroxylase is identical to factor inhibiting HIF (FIH) and is related to the cupin structural family. J. Biol. Chem. 277, 26351–26355. 10.1074/jbc.C200273200 12042299

[B60] Hirota K. Semenza G. L. (2001). Rac1 activity is required for the activation of hypoxia-inducible factor 1. J. Biol. Chem. 276, 21166–21172. 10.1074/jbc.M100677200 11283021

[B61] Hoeflich K. P. Luo J. Rubie E. A. Tsao M. S. Jin O. Woodgett J. R. (2000). Requirement for glycogen synthase kinase-3β in cell survival and NF-κB activation. Nature 406, 86–90. 10.1038/35017574 10894547

[B62] Hogenesch J. B. Gu Y. Z. Jain S. Bradfield C. A. (1998). The basic-helix-loop-helix-PAS orphan MOP3 forms transcriptionally active complexes with circadian and hypoxia factors. Proc. Natl. Acad. Sci. U.S.A. 95, 5474–5479. 10.1073/pnas.95.10.5474 9576906 PMC20401

[B63] Hu C. J. Wang L. Y. Chodosh L. A. Keith B. Simon M. C. (2003). Differential roles of hypoxia-inducible factor 1α (HIF-1α) and HIF-2α in hypoxic gene regulation. Mol. Cell. Biol. 23, 9361–9374. 10.1128/MCB.23.24.9361-9374.2003 14645546 PMC309606

[B64] Huang L. E. Gu J. Schau M. Bunn H. F. (1998). Regulation of hypoxia-inducible factor 1α is mediated by an O2-dependent degradation domain via the ubiquitin-proteasome pathway. Proc. Natl. Acad. Sci. U.S.A. 95, 7987–7992. 10.1073/pnas.95.14.7987 9653127 PMC20916

[B65] Hubert A. Paris S. Piret J. P. Ninane N. Raes M. Michiels C. (2006). Casein kinase 2 inhibition decreases hypoxia-inducible factor-1 activity under hypoxia through elevated p53 protein level. J. Cell. Sci. 119, 3351–3362. 10.1242/jcs.03069 16882692

[B66] Hudson C. C. Liu M. Chiang G. G. Otterness D. M. Loomis D. C. Kaper F.. (2002). Regulation of hypoxia-inducible factor 1α expression and function by the mammalian target of rapamycin. Mol. Cell. Biol. 22, 7004–7014. 10.1128/MCB.22.20.7004-7014.2002 12242281 PMC139825

[B67] Hur E. Chang K. Y. Lee E. Lee S. K. Park H. (2001). Mitogen-activated protein kinase kinase inhibitor PD98059 blocks the trans-activation but not the stabilization or DNA binding ability of hypoxia-inducible factor-1α. Mol. Pharmacol. 59, 1216–1224. 10.1124/mol.59.5.1216 11306706

[B68] Hwang A. B. Ryu E. A. Artan M. Chang H. W. Kabir M. H. Nam H. J.. (2014). Feedback regulation via AMPK and HIF-1 mediates ROS-dependent longevity in *Caenorhabditis elegans*. Proc. Natl. Acad. Sci. U.S.A. 111, E4458–E4467. 10.1073/pnas.1411199111 25288734 PMC4210294

[B69] Hwang J. T. Lee M. Jung S. N. Lee H. J. Kang I. Kim S. S.. (2004). AMP-activated protein kinase activity is required for vanadate-induced hypoxia-inducible factor 1α expression in DU145 cells. Carcinogenesis 25, 2497–2507. 10.1093/carcin/bgh253 15297373

[B70] Inoki K. Corradetti M. N. Guan K. L. (2005). Dysregulation of the TSC-mTOR pathway in human disease. Nat. Genet. 37, 19–24. 10.1038/ng1494 15624019

[B71] Irigoyen J. P. Munoz-Canoves P. Montero L. Koziczak M. Nagamine Y. (1999). The plasminogen activator system: biology and regulation. Cell Mol. Life Sci. 56, 104–132. 10.1007/PL00000615 11213252 PMC11146966

[B72] Jiang B. H. Zheng J. Z. Leung S. W. Roe R. Semenza G. L. (1997). Transactivation and inhibitory domains of hypoxia-inducible factor 1α. Modulation of transcriptional activity by oxygen tension. J. Biol. Chem. 272, 19253–19260. 10.1074/jbc.272.31.19253 9235919

[B73] Jung S. N. Yang W. K. Kim J. Kim H. S. Kim E. J. Yun H.. (2008). Reactive oxygen species stabilize hypoxia-inducible factor-1 α protein and stimulate transcriptional activity via AMP-activated protein kinase in DU145 human prostate cancer cells. Carcinogenesis 29, 713–721. 10.1093/carcin/bgn032 18258605

[B74] Kaelin W. G. (2005). Proline hydroxylation and gene expression. Annu. Rev. Biochem. 74, 115–128. 10.1146/annurev.biochem.74.082803.133142 15952883

[B75] Kaelin W. G. Jr. (2011). Cancer and altered metabolism: potential importance of hypoxia-inducible factor and 2-oxoglutarate-dependent dioxygenases. Cold Spring Harb. Symp. Quant. Biol. 76, 335–345. 10.1101/sqb.2011.76.010975 22089927 PMC4197849

[B76] Kallio P. J. Okamoto K. O'Brien S. Carrero P. Makino Y. Tanaka H.. (1998). Signal transduction in hypoxic cells: inducible nuclear translocation and recruitment of the CBP/p300 coactivator by the hypoxia-inducible factor-1α. EMBO J. 17, 6573–6586. 10.1093/emboj/17.22.6573 9822602 PMC1171004

[B77] Kaluz S. Kaluzova M. Stanbridge E. J. (2006). The role of extracellular signal-regulated protein kinase in transcriptional regulation of the hypoxia marker carbonic anhydrase IX. J. Cell. Biochem. 97, 207–216. 10.1002/jcb.20633 16270297

[B78] Kerkela R. Kockeritz L. Macaulay K. Zhou J. Doble B. W. Beahm C.. (2008). Deletion of GSK-3β in mice leads to hypertrophic cardiomyopathy secondary to cardiomyoblast hyperproliferation. J. Clin. Invest. 118, 3609–3618. 10.1172/JCI36245 18830417 PMC2556242

[B79] Kietzmann T. (2009). The hypoxia-inducible factor (HIF) pathway as a target for prevention and treatment of clinical manifestations. Curr. Pharm. Des. 15, 3837–3838. 10.2174/138161209789649385 19925432

[B80] Kietzmann T. (2010). Intracellular redox compartments: mechanisms and significances. Antioxid. Redox Signal. 13, 395–398. 10.1089/ars.2009.3001 20136596

[B81] Kietzmann T. Cornesse Y. Brechtel K. Modaressi S. Jungermann K. (2001). Perivenous expression of the mRNA of the three hypoxia-inducible factor α-subunits, HIF1α, HIF2α and HIF3α, in rat liver. Biochem. J. 354, 531–537. 10.1042/bj3540531 11237857 PMC1221684

[B82] Kietzmann T. Gorlach A. (2005). Reactive oxygen species in the control of hypoxia-inducible factor-mediated gene expression. Semin. Cell Dev. Biol. 16, 474–486. 10.1016/j.semcdb.2005.03.010 15905109

[B83] Kim D. H. Sarbassov D. D. Ali S. M. Latek R. R. Guntur K. V. Erdjument-Bromage H.. (2003). GβL, a positive regulator of the rapamycin-sensitive pathway required for the nutrient-sensitive interaction between raptor and mTOR. Mol. Cell 11, 895–904. 10.1016/S1097-2765(03)00114-X 12718876

[B84] Kim Y. S. Ahn K. H. Kim S. Y. Jeong J. W. (2009). Okadaic acid promotes angiogenesis via activation of hypoxia-inducible factor-1. Cancer Lett. 276, 102–108. 10.1016/j.canlet.2008.10.034 19054610

[B85] Koritzinsky M. Magagnin M. G. van den Beucken T. Seigneuric R. Savelkouls K. Dostie J.. (2006). Gene expression during acute and prolonged hypoxia is regulated by distinct mechanisms of translational control. EMBO J. 25, 1114–1125. 10.1038/sj.emboj.7600998 16467844 PMC1409715

[B86] Kruiswijk F. Labuschagne C. F. Vousden K. H. (2015). P53 in survival, death and metabolic health: a lifeguard with a licence to kill. Nat. Rev. Mol. Cell Biol. 16, 393–405. 10.1038/nrm4007 26122615

[B87] Kwon S. J. Song J. J. Lee Y. J. (2005). Signal pathway of hypoxia-inducible factor-1α phosphorylation and its interaction with von Hippel-Lindau tumor suppressor protein during ischemia in MiaPaCa-2 pancreatic cancer cells. Clin. Cancer Res. 11, 7607–7613. 10.1158/1078-0432.CCR-05-0981 16278378

[B88] Land S. C. Tee A. R. (2007). Hypoxia-inducible factor 1α is regulated by the mammalian target of rapamycin (mTOR) via an mTOR signaling motif. J. Biol. Chem. 282, 20534–20543. 10.1074/jbc.M611782200 17502379

[B89] Lando D. Peet D. J. Gorman J. J. Whelan D. A. Whitelaw M. L. Bruick R. K. (2002). FIH-1 is an asparaginyl hydroxylase enzyme that regulates the transcriptional activity of hypoxia-inducible factor. Genes Dev. 16, 1466–1471. 10.1101/gad.991402 12080085 PMC186346

[B90] Laughner E. Taghavi P. Chiles K. Mahon P. C. Semenza G. L. (2001). HER2 (neu) signaling increases the rate of hypoxia-inducible factor 1α (HIF-1α) synthesis: novel mechanism for HIF-1-mediated vascular endothelial growth factor expression. Mol. Cell. Biol. 21, 3995–4004. 10.1128/MCB.21.12.3995-4004.2001 11359907 PMC87062

[B91] Lee E. Yim S. Lee S. K. Park H. (2002). Two transactivation domains of hypoxia-inducible factor-1α regulated by the MEK-1/p42/p44 MAPK pathway. Mol. Cells 14, 9–15. 12243358

[B92] Lee M. Hwang J. T. Lee H. J. Jung S. N. Kang I. Chi S. G.. (2003). AMP-activated protein kinase activity is critical for hypoxia-inducible factor-1 transcriptional activity and its target gene expression under hypoxic conditions in DU145 cells. J. Biol. Chem. 278, 39653–39661. 10.1074/jbc.M306104200 12900407

[B93] Li M. Wang X. Meintzer M. K. Laessig T. Birnbaum M. J. Heidenreich K. A. (2000). Cyclic AMP promotes neuronal survival by phosphorylation of glycogen synthase kinase 3β. Mol. Cell. Biol. 20, 9356–9363. 10.1128/MCB.20.24.9356-9363.2000 11094086 PMC102192

[B94] Li Y. Inoki K. Vacratsis P. Guan K. L. (2003). The p38 and MK2 kinase cascade phosphorylates tuberin, the tuberous sclerosis 2 gene product, and enhances its interaction with 14-3-3. J. Biol. Chem. 278, 13663–13671. 10.1074/jbc.M300862200 12582162

[B95] Li Z. Na X. Wang D. Schoen S. R. Messing E. M. Wu G. (2002a). Ubiquitination of a novel deubiquitinating enzyme requires direct binding to von Hippel-Lindau tumor suppressor protein. J. Biol. Chem. 277, 4656–4662. 10.1074/jbc.M108269200 11739384

[B96] Li Z. Wang D. Messing E. M. Wu G. (2005). VHL protein-interacting deubiquitinating enzyme 2 deubiquitinates and stabilizes HIF-1α. EMBO Rep. 6, 373–378. 10.1038/sj.embor.7400377 15776016 PMC1299287

[B97] Li Z. Wang D. Na X. Schoen S. R. Messing E. M. Wu G. (2002b). Identification of a deubiquitinating enzyme subfamily as substrates of the von Hippel-Lindau tumor suppressor. Biochem. Biophys. Res. Commun. 294, 700–709. 10.1016/S0006-291X(02)00534-X 12056827

[B98] Love K. R. Catic A. Schlieker C. Ploegh H. L. (2007). Mechanisms, biology and inhibitors of deubiquitinating enzymes. Nat. Chem. Biol. 3, 697–705. 10.1038/nchembio.2007.43 17948018

[B99] MacAulay K. Doble B. W. Patel S. Hansotia T. Sinclair E. M. Drucker D. J.. (2007). Glycogen synthase kinase 3α-specific regulation of murine hepatic glycogen metabolism. Cell. Metab. 6, 329–337. 10.1016/j.cmet.2007.08.013 17908561

[B100] Mahon P. C. Hirota K. Semenza G. L. (2001). FIH-1: a novel protein that interacts with HIF-1α and VHL to mediate repression of HIF-1 transcriptional activity. Genes Dev. 15, 2675–2686. 10.1101/gad.924501 11641274 PMC312814

[B101] Makino Y. Uenishi R. Okamoto K. Isoe T. Hosono O. Tanaka H.. (2007). Transcriptional up-regulation of inhibitory PAS domain protein gene expression by hypoxia-inducible factor 1 (HIF-1): a negative feedback regulatory circuit in HIF-1-mediated signaling in hypoxic cells. J. Biol. Chem. 282, 14073–14082. 10.1074/jbc.M700732200 17355974

[B102] Malumbres M. (2014). Cyclin-dependent kinases. Genome Biol. 15, 122. 10.1186/gb4184 25180339 PMC4097832

[B103] Malumbres M. (2015). Keeping order in anaphase. Dev. Cell. 35, 403–404. 10.1016/j.devcel.2015.11.011 26609955

[B104] Malumbres M. Harlow E. Hunt T. Hunter T. Lahti J. M. Manning G.. (2009). Cyclin-dependent kinases: a family portrait. Nat. Cell Biol. 11, 1275–1276. 10.1038/ncb1109-1275 19884882 PMC2914104

[B105] Masson N. Ratcliffe P. J. (2014). Hypoxia signaling pathways in cancer metabolism: the importance of co-selecting interconnected physiological pathways. Cancer Metab. 2, 3–20. 10.1186/2049-3002-2-3 24491179 PMC3938304

[B106] Mazure N. M. Chen E. Y. Laderoute K. R. Giaccia A. J. (1997). Induction of vascular endothelial growth factor by hypoxia is modulated by a phosphatidylinositol 3-kinase/Akt signaling pathway in Ha-ras-transformed cells through a hypoxia inducible factor-1 transcriptional element. Blood 90, 3322–3331. 9345014

[B107] McKinsey T. A. Zhang C. L. Olson E. N. (2001). Identification of a signal-responsive nuclear export sequence in class II histone deacetylases. Mol. Cell. Biol. 21, 6312–6321. 10.1128/MCB.21.18.6312-6321.2001 11509672 PMC87361

[B108] Minet E. Arnould T. Michel G. Roland I. Mottet D. Raes M.. (2000). ERK activation upon hypoxia: involvement in HIF-1 activation. FEBS Lett. 468, 53–58. 10.1016/S0014-5793(00)01181-9 10683440

[B109] Montenarh M. (2010). Cellular regulators of protein kinase CK2. Cell Tissue Res. 342, 139–146. 10.1007/s00441-010-1068-3 20976471

[B110] Moore P. A. Rosen C. A. Carter K. C. (1996). Assignment of the human FKBP12-rapamycin-associated protein (FRAP) gene to chromosome 1p36 by fluorescence *in situ* hybridization. Genomics 33, 331–332. 10.1006/geno.1996.0206 8660990

[B111] Morrison D. K. Davis R. J. (2003). Regulation of MAP kinase signaling modules by scaffold proteins in mammals. Annu. Rev. Cell Dev. Biol. 19, 91–118. 10.1146/annurev.cellbio.19.111401.091942 14570565

[B112] Mottet D. Dumont V. Deccache Y. Demazy C. Ninane N. Raes M.. (2003). Regulation of hypoxia-inducible factor-1α protein level during hypoxic conditions by the phosphatidylinositol 3-kinase/Akt/glycogen synthase kinase 3β pathway in HepG2 cells. J. Biol. Chem. 278, 31277–31285. 10.1074/jbc.M300763200 12764143

[B113] Mottet D. Ruys S. P. D. Demazy C. Raes M. Michiels C. (2005). Role for casein kinase 2 in the regulation of HIF-1 activity. Int. J. Cancer 117, 764–774. 10.1002/ijc.21268 15957168

[B114] Mukai F. Ishiguro K. Sano Y. Fujita S. C. (2002). Alternative splicing isoform of tau protein kinase I/glycogen synthase kinase 3β. J. Neurochem. 81, 1073–1083. 10.1046/j.1471-4159.2002.00918.x 12065620

[B115] Myer D. L. Robbins S. B. Yin M. Boivin G. P. Liu Y. Greis K. D.. (2011). Absence of polo-like kinase 3 in mice stabilizes Cdc25A after DNA damage but is not sufficient to produce tumors. Mutat. Res. 714, 1–10. 10.1016/j.mrfmmm.2011.02.006 21376736 PMC7364384

[B116] Mylonis I. Chachami G. Paraskeva E. Simos G. (2008). Atypical CRM1-dependent nuclear export signal mediates regulation of hypoxia-inducible factor-1α by MAPK. J. Biol. Chem. 283, 27620–27627. 10.1074/jbc.M803081200 18687685

[B117] Mylonis I. Chachami G. Samiotaki M. Panayotou G. Paraskeva E. Kalousi A.. (2006). Identification of MAPK phosphorylation sites and their role in the localization and activity of hypoxia-inducible factor-1α. J. Biol. Chem. 281, 33095–33106. 10.1074/jbc.M605058200 16954218

[B118] Niefind K. Raaf J. Issinger O. G. (2009). Protein kinase CK2 in health and disease: protein kinase CK2: from structures to insights. Cell Mol. Life Sci. 66, 1800–1816. 10.1007/s00018-009-9149-8 19387553 PMC11115703

[B119] Nijman S. M. Luna-Vargas M. P. Velds A. Brummelkamp T. R. Dirac A. M. Sixma T. K.. (2005). A genomic and functional inventory of deubiquitinating enzymes. Cell 123, 773–786. 10.1016/j.cell.2005.11.007 16325574

[B120] Olson J. M. Hallahan A. R. (2004). p38 MAP kinase: a convergence point in cancer therapy. Trends Mol. Med. 10, 125–129. 10.1016/j.molmed.2004.01.007 15102355

[B121] Pasanen A. Heikkila M. Rautavuoma K. Hirsila M. Kivirikko K. I. Myllyharju J. (2010). Hypoxia-inducible factor (HIF)-3α is subject to extensive alternative splicing in human tissues and cancer cells and is regulated by HIF-1 but not HIF-2. Int. J. Biochem. Cell Biol. 42, 1189–1200. 10.1016/j.biocel.2010.04.008 20416395

[B122] Raman M. Chen W. Cobb M. H. (2007). Differential regulation and properties of MAPKs. Oncogene 26, 3100–3112. 10.1038/sj.onc.1210392 17496909

[B123] Richard D. E. Berra E. Gothie E. Roux D. Pouyssegur J. (1999). p42/p44 mitogen-activated protein kinases phosphorylate hypoxia-inducible factor 1α (HIF-1α) and enhance the transcriptional activity of HIF-1. J. Biol. Chem. 274, 32631–32637. 10.1074/jbc.274.46.32631 10551817

[B124] Rincon M. Davis R. J. (2009). Regulation of the immune response by stress-activated protein kinases. Immunol. Rev. 228, 212–224. 10.1111/j.1600-065X.2008.00744.x 19290930

[B125] Roh M. S. Eom T. Y. Zmijewska A. A. De Sarno P. Roth K. A. Jope R. S. (2005). Hypoxia activates glycogen synthase kinase-3 in mouse brain *in vivo*: protection by mood stabilizers and imipramine. Biol. Psychiatry 57, 278–286. 10.1016/j.biopsych.2004.10.039 15691529

[B126] Russo A. A. Jeffrey P. D. Pavletich N. P. (1996). Structural basis of cyclin-dependent kinase activation by phosphorylation. Nat. Struct. Biol. 3, 696–700. 10.1038/nsb0896-696 8756328

[B127] Sabio G. Davis R. J. (2014). TNF and MAP kinase signalling pathways. Semin. Immunol. 26, 237–245. 10.1016/j.smim.2014.02.009 24647229 PMC4099309

[B128] Saeki K. Machida M. Kinoshita Y. Takasawa R. Tanuma S. (2011). Glycogen synthase kinase-3β2 has lower phosphorylation activity to tau than glycogen synthase kinase-3β1. Biol. Pharm. Bull. 34, 146–149. 10.1248/bpb.34.146 21212533

[B129] Salceda S. Beck I. Srinivas V. Caro J. (1997). Complex role of protein phosphorylation in gene activation by hypoxia. Kidney Int. 51, 556–559. 10.1038/ki.1997.78 9027738

[B130] Sang N. Stiehl D. P. Bohensky J. Leshchinsky I. Srinivas V. Caro J. (2003). MAPK signaling up-regulates the activity of hypoxia-inducible factors by its effects on p300. J. Biol. Chem. 278, 14013–14019. 10.1074/jbc.M209702200 12588875 PMC4518846

[B131] Sarbassov D. D. Ali S. M. Kim D. H. Guertin D. A. Latek R. R. Erdjument-Bromage H.. (2004). Rictor, a novel binding partner of mTOR, defines a rapamycin-insensitive and raptor-independent pathway that regulates the cytoskeleton. Curr. Biol. 14, 1296–1302. 10.1016/j.cub.2004.06.054 15268862

[B132] Scheel H. Hofmann K. (2005). Prediction of a common structural scaffold for proteasome lid, COP9-signalosome and eIF3 complexes. BMC Bioinformatics 6:71. 10.1186/1471-2105-6-71 15790418 PMC1274264

[B133] Schnitzer S. E. Schmid T. Zhou J. Eisenbrand G. Brune B. (2005). Inhibition of GSK3β by indirubins restores HIF-1α accumulation under prolonged periods of hypoxia/anoxia. FEBS Lett. 579, 529–533. 10.1016/j.febslet.2004.12.023 15642371

[B134] Scortegagna M. Ding K. Oktay Y. Gaur A. Thurmond F. Yan L. J.. (2003). Multiple organ pathology, metabolic abnormalities and impaired homeostasis of reactive oxygen species in Epas1-/- mice. Nat. Genet. 35, 331–340. 10.1038/ng1266 14608355

[B135] Semenza G. L. (2003). Targeting HIF-1 for cancer therapy. Nat. Rev. Cancer. 3, 721–732. 10.1038/nrc1187 13130303

[B136] Serviddio G. Bellanti F. Vendemiale G. (2013). Free radical biology for medicine: learning from nonalcoholic fatty liver disease. Free Radic. Biol. Med. 65, 952–968. 10.1016/j.freeradbiomed.2013.08.174 23994574

[B137] Shiloh Y. Ziv Y. (2013). The ATM protein kinase: regulating the cellular response to genotoxic stress, and more. Nat. Rev. Mol. Cell Biol. 14, 197–210. 10.1038/nrm3546 23847781

[B138] Shoshani T. Faerman A. Mett I. Zelin E. Tenne T. Gorodin S.. (2002). Identification of a novel hypoxia-inducible factor 1-responsive gene, RTP801, involved in apoptosis. Mol. Cell. Biol. 22, 2283–2293. 10.1128/mcb.22.7.2283-2293.2002 11884613 PMC133671

[B139] Skinner H. D. Zhong X. S. Gao N. Shi X. Jiang B. H. (2004). Arsenite induces p70S6K1 activation and HIF-1α expression in prostate cancer cells. Mol. Cell. Biochem. 255, 19–23. 10.1023/B:MCBI.0000007257.67733.3b 14971642

[B140] Sodhi A. Montaner S. Miyazaki H. Gutkind J. S. (2001). MAPK and Akt act cooperatively but independently on hypoxia inducible factor-1α in rasV12 upregulation of VEGF. Biochem. Biophys. Res. Commun. 287, 292–300. 10.1006/bbrc.2001.5532 11549290

[B141] Sodhi A. Montaner S. Patel V. Zohar M. Bais C. Mesri E. A.. (2000). The Kaposi's sarcoma-associated herpes virus G protein-coupled receptor up-regulates vascular endothelial growth factor expression and secretion through mitogen-activated protein kinase and p38 pathways acting on hypoxia-inducible factor 1α. Cancer Res. 60, 4873–4880. 10987301

[B142] Sowter H. M. Raval R. R. Moore J. W. Ratcliffe P. J. Harris A. L. (2003). Predominant role of hypoxia-inducible transcription factor (Hif)-1α versus Hif-2α in regulation of the transcriptional response to hypoxia. Cancer Res. 63, 6130–6134. 14559790

[B143] St-Denis N. A. Litchfield D. W. (2009). Protein kinase CK2 in health and disease: from birth to death: the role of protein kinase CK2 in the regulation of cell proliferation and survival. Cell Mol. Life Sci. 66, 1817–1829. 10.1007/s00018-009-9150-2 19387552 PMC11115660

[B144] Sutendra G. Dromparis P. Kinnaird A. Stenson T. H. Haromy A. Parker J. M.. (2013). Mitochondrial activation by inhibition of PDKII suppresses HIF1a signaling and angiogenesis in cancer. Oncogene 32, 1638–1650. 10.1038/onc.2012.198 22614004

[B145] Suzuki H. Tomida A. Tsuruo T. (2001). Dephosphorylated hypoxia-inducible factor 1α as a mediator of p53-dependent apoptosis during hypoxia. Oncogene 20, 5779–5788. 10.1038/sj.onc.1204742 11593383

[B146] Thomas G. V. Tran C. Mellinghoff I. K. Welsbie D. S. Chan E. Fueger B.. (2006). Hypoxia-inducible factor determines sensitivity to inhibitors of mTOR in kidney cancer. Nat. Med. 12, 122–127. 10.1038/nm1337 16341243

[B147] Tian H. McKnight S. L. Russell D. W. (1997). Endothelial PAS domain protein 1 (EPAS1), a transcription factor selectively expressed in endothelial cells. Genes Dev. 11, 72–82. 10.1101/gad.11.1.72 9000051

[B148] Toffoli S. Feron O. Raes M. Michiels C. (2007). Intermittent hypoxia changes HIF-1α phosphorylation pattern in endothelial cells: unravelling of a new PKA-dependent regulation of HIF-1α. Biochim. Biophys. Acta 1773, 1558–1571. 10.1016/j.bbamcr.2007.06.002 17662481

[B149] Tormos A. M. Talens-Visconti R. Nebreda A. R. Sastre J. (2013). p38 MAPK: a dual role in hepatocyte proliferation through reactive oxygen species. Free Radic. Res. 47, 905–916. 10.3109/10715762.2013.821200 23906070

[B150] Toschi A. Lee E. Gadir N. Ohh M. Foster D. A. (2008). Differential dependence of hypoxia-inducible factors 1 α and 2 α on mTORC1 and mTORC2. J. Biol. Chem. 283, 34495–34499. 10.1074/jbc.C800170200 18945681 PMC2596400

[B151] Treins C. Giorgetti-Peraldi S. Murdaca J. Semenza G. L. Van Obberghen E. (2002). Insulin stimulates hypoxia-inducible factor 1 through a phosphatidylinositol 3-kinase/target of rapamycin-dependent signaling pathway. J. Biol. Chem. 277, 27975–27981. 10.1074/jbc.M204152200 12032158

[B152] Triantafyllou A. Liakos P. Tsakalof A. Georgatsou E. Simos G. Bonanou S. (2006). Cobalt induces hypoxia-inducible factor-1α (HIF-1α) in HeLa cells by an iron-independent, but ROS-, PI-3K- and MAPK-dependent mechanism. Free Radic. Res. 40, 847–856. 10.1080/10715760600730810 17015263

[B153] Wang G. L. Jiang B. H. Rue E. A. Semenza G. L. (1995a). Hypoxia-inducible factor 1 is a basic-helix-loop-helix-PAS heterodimer regulated by cellular O2 tension. Proc. Natl. Acad. Sci. U.S.A. 92, 5510–5514. 10.1073/pnas.92.12.5510 7539918 PMC41725

[B154] Wang G. L. Jiang B. H. Semenza G. L. (1995b). Effect of protein kinase and phosphatase inhibitors on expression of hypoxia-inducible factor 1. Biochem. Biophys. Res. Commun. 216, 669–675. 10.1006/bbrc.1995.2674 7488163

[B155] Warfel N. A. Dolloff N. G. Dicker D. T. Malysz J. El-Deiry W. S. (2013). CDK1 stabilizes HIF-1α via direct phosphorylation of Ser668 to promote tumor growth. Cell. Cycle 12, 3689–3701. 10.4161/cc.26930 24189531 PMC3903720

[B156] Watanabe N. Arai H. Iwasaki J. Shiina M. Ogata K. Hunter T.. (2005). Cyclin-dependent kinase (CDK) phosphorylation destabilizes somatic Wee1 via multiple pathways. Proc. Natl. Acad. Sci. U.S.A. 102, 11663–11668. 10.1073/pnas.0500410102 16085715 PMC1187955

[B157] Wenger R. H. (2002). Cellular adaptation to hypoxia: O2-sensing protein hydroxylases, hypoxia-inducible transcription factors, and O2-regulated gene expression. FASEB J. 16, 1151–1162. 10.1096/fj.01-0944rev 12153983

[B158] Wenger R. H. Stiehl D. P. Camenisch G. (2005). Integration of oxygen signaling at the consensus HRE. Sci. STKE 2005:re12. 10.1126/stke.3062005re12 16234508

[B159] Wullschleger S. Loewith R. Hall M. N. (2006). TOR signaling in growth and metabolism. Cell 124, 471–484. 10.1016/j.cell.2006.01.016 16469695

[B160] Xu D. Yao Y. Lu L. Costa M. Dai W. (2010). Plk3 functions as an essential component of the hypoxia regulatory pathway by direct phosphorylation of HIF-1α. J. Biol. Chem. 285, 38944–38950. 10.1074/jbc.M110.160325 20889502 PMC2998109

[B161] Yang Y. Bai J. Shen R. Brown S. A. Komissarova E. Huang Y.. (2008). Polo-like kinase 3 functions as a tumor suppressor and is a negative regulator of hypoxia-inducible factor-1 α under hypoxic conditions. Cancer Res. 68, 4077–4085. 10.1158/0008-5472.CAN-07-6182 18519666 PMC3725591

[B162] Zhong H. Chiles K. Feldser D. Laughner E. Hanrahan C. Georgescu M. M.. (2000). Modulation of hypoxia-inducible factor 1α expression by the epidermal growth factor/phosphatidylinositol 3-kinase/PTEN/AKT/FRAP pathway in human prostate cancer cells: implications for tumor angiogenesis and therapeutics. Cancer Res. 60, 1541–1545. 10749120

[B163] Zundel W. Schindler C. Haas-Kogan D. Koong A. Kaper F. Chen E.. (2000). Loss of PTEN facilitates HIF-1-mediated gene expression. Genes Dev. 14, 391–396. 10.1101/gad.14.4.391 10691731 PMC316386

